# African swine fever virus transmembrane protein pEP84R guides core assembly

**DOI:** 10.1371/journal.ppat.1011136

**Published:** 2023-01-30

**Authors:** Alí Alejo, Mayte García-Castey, Milagros Guerra, Bruno Hernáez, Verónica Martín, Tania Matamoros, Germán Andrés

**Affiliations:** 1 Centro de Investigación en Sanidad Animal; Instituto Nacional de Investigación y Tecnología Agraria y Alimentaria, Consejo Superior de Investigaciones Científicas, Valdeolmos, Madrid, Spain; 2 Centro de Biología Molecular Severo Ochoa; Consejo Superior de Investigaciones Científicas and Universidad Autónoma de Madrid, Madrid, Spain; Institute for Animal Health, Pirbright Laboratory, UNITED KINGDOM

## Abstract

African swine fever virus (ASFV) causes a devastating hemorrhagic disease with worldwide circulation and no widely available therapeutic prevention. The infectious particle has a multilayered architecture that is articulated upon an endoplasmic reticulum (ER)-derived inner envelope. This membrane acts as docking platform for the assembly of the outer icosahedral capsid and the underlying core shell, a bridging layer required for the formation of the central genome-containing nucleoid. While the details of outer capsid assembly are relatively well understood, those of core formation remain unclear. Here we report the functional characterization of pEP84R, a transmembrane polypeptide embedded in the inner envelope that surrounds the viral core. Using an ASFV recombinant inducibly expressing the *EP84R* gene, we show that absence of pEP84R results in the formation of non-infectious core-less icosahedral particles displaying a significant DNA-packaging defect. Concomitantly, aberrant core shell-like structures formed by co-assembly of viral polyproteins pp220 and pp62 are mistargeted to non-ER membranes, as also occurs when these are co-expressed in the absence of other viral proteins. Interestingly, co-expression of both polyproteins with pEP84R led to the formation of ER-targeted core shell-like assemblies and co-immunoprecipitation assays showed that pEP84R binds to the N-terminal region of pp220. Altogether, these results indicate that pEP84R plays a crucial role in core assembly by targeting the core shell polyproteins to the inner viral envelope, which enables subsequent genome packaging and nucleoid formation. These findings unveil a key regulatory mechanism for ASFV morphogenesis and identify a relevant novel target for the development of therapeutic tools against this re-emerging threat.

## Author summary

African swine fever virus is a complex multilayered DNA virus that produces a highly lethal disease that currently affects domestic and wild pigs in Africa, Europe, Asia, Oceania and more recently the Americas. In spite of extensive research, widely available vaccines or any other antiviral strategy are still lacking and relevant questions on ASFV replication remain to be addressed. The present study characterizes the viral membrane protein pEP84R as a key component for the virion assembly. Using genetical, biochemical and microscopic approaches, we provide evidence that pEP84R acts as a molecular link that bridges the internal genome-containing core to the external viral layers. These findings significantly expand our understanding of the mechanisms underlying the formation of the infectious virus particle, which may be relevant for vaccine and antiviral research.

## Introduction

African swine fever virus (ASFV) causes a devastating hemorrhagic disease in both domestic pigs and Eurasian wild boars, whose control is currently based on culling and strict containment measures due to the absence of widely available vaccine or other therapeutic tools available [1–5]. The disease, which is endemic in sub-Saharan Africa, has rapidly spread over the last years across many countries of Europe, Asia, and Oceania, reaching very recently the Americas (Dominican Republic and Haiti). Currently, African swine fever virus represents one of the major threats for the global swine industry and food safety.

ASFV belongs to the family *Asfarviridae*, within the recently established Phylum Nucleocytoviricota [6], a monophyletic clade of large and giant DNA eukaryotic viruses [7]. Previously known as nucleocytoplasmic large DNA viruses (NCLDV), Nucleocytoviricota are ubiquitous in the environment and have been found to infect very diverse hosts from all over the taxonomic range, notably heterotrophic unicellular eukaryotes, amoebae and several animal species including insects, amphibians, reptiles and mammals among others. Besides wild and domestic pigs, ASFV also infects soft ticks of the genus Ornithodoros, being the only DNA arbovirus known to date.

The infectious ASFV particle, with an icosahedral morphology and a diameter of about 250 nm between opposite vertices, possesses a multilayered architecture that comprises a central genome-containing nucleoid that is successively enclosed by a thick protein core shell, an inner lipoprotein membrane, an outer protein capsid, and an external lipoprotein membrane [8–12]. The ASFV genome is a 170–190 kbp long dsDNA molecule comprising more than 150 genes, about half of which encode virion proteins [13].

ASFV primarily infects monocytes and macrophages in the porcine host, where it deploys a tightly regulated transcriptional programme including several different gene categories [14, 15]. As in other Nucleocytoviricota, ASFV genome replication and morphogenesis take place within perinuclear cytoplasmic areas termed viral factories [16–18]. At the ultrastructural level, ASFV assembly commences with the appearance within the virus factory of membrane fragments, which are thought to be derived from the endoplasmic reticulum (ER) through a poorly understood process [18–21]. These precursor membranes give rise to the inner viral envelope, which represents a central element of virus assembly as it coordinates the structured formation of the outer capsid and the underlying core shell on opposing surfaces. Subsequently, the resulting icosahedral intermediates enclose the genome-containing material to form the central nucleoid [16]. Finally, the intracellular full particles move to the plasma membrane (PM), where they egress by budding, acquiring an additional lipid envelope [22] that is not strictly necessary for infectivity [23].

Recent elucidation of the ASFV architecture by cryo-electron microscopy (cryo-EM) has provided a detailed map of the outer capsid structure, which consists of a hexagonal lattice (triangulation number T = 277) composed of 2,760 trimeric capsomers of the major capsid protein (MCP) p72, and of 60 copies of a penton complex, which is formed by the minor proteins p49 and pH240R, at the vertices [8, 11, 12]. Interestingly, the anchorage of the outer capsid to the inner envelope mainly occurs through the interaction of the MCP p72 with the major inner envelope protein p17 [11, 12].

Less is known about the molecular organization of the viral core and its interaction with the inner envelope. The internal nucleoid contains the viral genome along with the major nucleoproteins pK78R and pA104R and, possibly, a set of viral enzymes and transcription factors involved in viral transcription, mRNA modification and DNA repair [13]. The nucleoid is linked to the inner membrane through the core shell, a matrix-like domain that results from the co-assembly of viral polyproteins pp220 and pp62 [9, 24]. Polyprotein processing by viral protease pS273R [25] gives rise to the structural products p5, p34, p14 and p150, derived from pp220 [13, 26], and p15, p35 and p8, which derive from pp62 [13, 27]. This late maturational step triggers structural rearrangements of the core shell and is crucial for the generation of infectious particles [28]. In this connection, the above mentioned cryo-EM studies have identified an internal icosahedral protein layer (T = 19) underneath the inner envelope that outlines the core shell of the mature ASFV particle [8, 12]. The nature of the polyprotein-derived mature products composing this inner capsid remains unknown due to insufficient resolution of the available three-dimensional reconstructions. At present, the details of viral core assembly, including its membrane anchoring, are largely unknown.

Here we show that the previously uncharacterized structural protein pEP84R is an inner envelope transmembrane protein which guides the assembly of the core shell by interacting with the polyprotein precursor pp220. This process, in turn, is critical for the formation of the genome-containing nucleoid and hence for the generation of infectious ASFV particles.

## Results

### ASFV structural protein pEP84R is a transmembrane protein of the inner viral envelope

**Fig 1:**
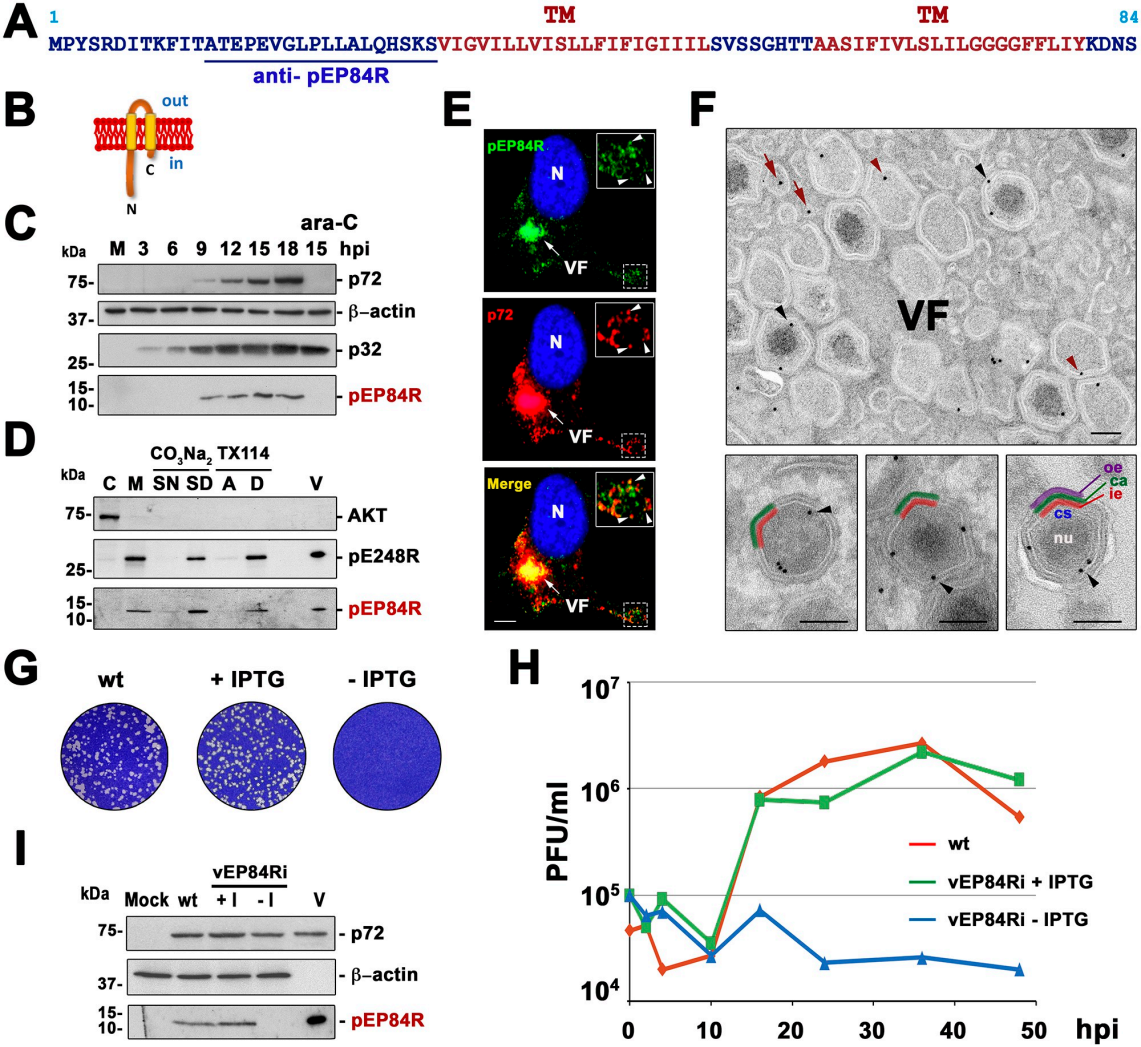
ASFV transmembrane protein pE84R is required for virus replication. **(A)** Amino acid (aa) sequence of pE84R protein in ASFV BA71V strain. The two putative transmembrane (TM) domains and the peptide sequence used to produce a rabbit anti-pEP84R antibody are indicated. **(B)** Predicted membrane topology of pEP84R. The N-terminal 30-aa region and the small C-terminal 4-aa tail are predicted to be facing the cytosol (in). **(C)** Time course of pEP84R expression. ASFV-infected Vero cells were dissociated at the indicated times and analyzed by western blotting for pEP84R along with the early protein p32, the late MCP p72 and ß-actin. Mock (M) and ASFV-infected cells incubated for 15 h in the presence of DNA synthesis inhibitor ara-C were also analyzed. Molecular masses (in kDa) are indicated. **(D)** Membrane-association of pEP84R protein. ASFV-infected cells were fractionated into cytosolic (C) and membrane/particulate (M) fraction. The latter was subjected to alkaline carbonate (CO_3_Na_2_) extraction or TX-114 phase separation to obtain supernatant (SN) and sediment (SD) fractions, or aqueous (A) and detergent-rich (D) phases, respectively. Equivalent amounts of each fraction along with purified ASFV (V) were analyzed by immunoblotting with antibodies to pEP84R, cytosolic marker AKT and transmembrane ASFV protein pE248R. **(E)** Subcellular localization of pEP84R. ASFV-infected cells were fixed at 18 hpi and labeled for pEP84R (green) and MCP p72 (red). Nucleus was stained with Hoechst 33258 (blue). Arrows indicate co-localization at viral factories (VF) while arrowheads in the insets indicate co-localization in scattered virus particles. Bar, 5 μm. **(F)** Subviral localization of pEP84R. ASFV-infected cells fixed as above were immunolabelled with anti-pEP84R antibody followed by protein A-gold (10 nm). Note that gold particles in immature (left) and mature (center) virus at the assembly sites, as well as mature extracellular virions (right), are detected at the interface between the core shell (cs, blue), and the inner envelope (ie, red layer). The DNA-containing nucleoid (nu, white), the outer capsid (ca, green) and outer envelope (oe, purple) are also indicated. Bars, 100 nm. **(G)** Plaque phenotype of vEP84Ri. Vero cell monolayers were infected with BA71V (wt) or vEP84Ri viruses in the presence (+) or absence (-) of IPTG. **(H)** One-step growth curves of vEP84Ri virus. Vero cells were infected with 5 pfu per cell of BA71V (wt) or vEP84Ri virus in the presence or absence of IPTG. At the indicated hpi the virus titer of each sample was determined. **(I)** Inducible expression of protein pEP84R. Vero cells were infected as indicated and lysates analyzed along with purified virus (V) by immunoblotting with antibodies to pEP84R, MCP p72 and ß-actin.

ASFV pEP84R is a small virion protein [13] with two putative transmembrane domains that is highly conserved among different ASFV strains (Fig 1A and S1 Fig). According to its predicted topology, most of its C-terminal half, which contains two 21 aa-long hydrophobic α-helical segments, would be embedded in the lipid bilayer while the N-terminal half (30 aa) and a short (4 aa) C-terminal tail would face the cytosol (Fig 1B).

The expression of pEP84R in ASFV-infected cells was analyzed by western blot with an anti-pE84R antibody raised against an N-terminal fragment (Fig 1A). As shown in Fig 1C, a specific 12-kDa protein band roughly corresponding to its expected size (9 kDa) was detected from 9 hours post infection (hpi) onwards, coinciding with the expression of MCP p72. This band was not detected in infected cells treated for 15 h with cytosine arabinoside (ara-C), an inhibitor of ASFV DNA replication and late transcription (Fig 1C), indicating that pEP84R is encoded by a late viral gene, in accordance with a previous transcriptomic analysis [14].

To assess the putative interaction of pEP84R with lipid membranes, cytosolic and membrane/particulate fractions of ASFV-infected cells were analyzed by western blotting. As shown in Fig 1D, protein pEP84R was mainly detected in the membrane fraction, as was transmembrane viral protein pE248R, but not cytosolic cellular kinase AKT. When the membrane fraction was subjected to alkaline carbonate extraction, a treatment that dissociates peripheral proteins from lipid membranes, pEP84R remained in the membrane sediment (Fig 1D). Moreover, it partitioned into the detergent phase after Triton X-114 treatment. In conclusion, pEP84R behaves as an integral membrane protein, in keeping with the membrane topology prediction.

To study the subcellular localization of pEP84R in the absence of other ASFV proteins, a Flag-tagged version was transiently expressed in Cos cells and analyzed by immunofluorescence. As shown in S2 Fig, pEP84R displayed a predominantly perinuclear pattern, which significantly colocalized with membrane markers for ER-Golgi intermediate compartment (ERGIC-53), cis-Golgi (GM130) and trans-Golgi network (TGN46), along with a weaker cytoplasmic reticular-like pattern emerging from the nuclear envelope, which colocalized with ER luminal marker protein disulfide isomerase (PDI). During ASFV infection, pEP84R signal was mainly detected within perinuclear viral factories, co-localizing with MCP p72 (Fig 1E). Additionally, pEP84R fluorescence was also detected in punctate structures scattered throughout the cytoplasm, which likely correspond to dispersed virus particles, as suggested by their partial co-localization with p72.

To analyze the subcellular distribution of pEP84R within the virus factories, immunoelectron microscopy (immuno-EM) was performed on cryosections of ASFV-infected Vero cells. As shown in Fig 1F **(upper panel)**, specific pEP84R labeling was detected in ER-derived precursor viral membranes as well as immature and mature icosahedral virus particles. Interestingly, immunogold labeling within the virus particles was mostly detected in close proximity to the interface between the viral core shell and the inner envelope (Fig 1F, **lower panels**) in both immature (left) and mature (middle) particles at the assembly sites as well as in mature budding particles at the PM (right).

Altogether, the predicted topology in combination with the biochemical and immunocytochemical evidence, indicate that pEP84R localizes in the inner envelope that encloses the viral core.

### Protein pEP84R is essential for ASFV replication

To investigate the role of protein pEP84R in viral replication, we generated a recombinant virus, vEP84Ri, derived from the BA71V strain, in which the expression of *EP84R* gene is under the control of an inducible, IPTG-dependent promoter (S3 Fig). Whole genome sequencing of vEP84Ri confirmed the absence of significant changes as compared to parental BA71V beyond the 2.7 kbp inducible cassette required for its construction.

As shown in Fig 1G, plaque formation by recombinant vEP84Ri in the presence of 1 mM IPTG was similar to that of the parental BA71V virus, while in its absence no lysis plaques were observed. Moreover, a one-step growth curve of vEP84Ri showed that under non-permissive conditions it failed to produce infectious virus, as titers were below those of the added inoculum at all time points (Fig 1H). By contrast, under permissive conditions, vEP84Ri produced similar titers to those of parental BA71V virus. Altogether, these results indicate that recombinant vEP84Ri behaves as an IPTG-dependent lethal conditional mutant.

To verify the inducible expression of pEP84R, Vero cells infected with parental BA71V or vEP84Ri virus in the absence or presence of IPTG were analyzed by Western blot. As shown in Fig 1I, while the expression levels of pEP84R were comparable in control and vEP84Ri permissive infections, pEP84R was virtually undetectable when vEP84Ri infection was conducted in the absence of IPTG. Overall, these results show that the conditional lethal phenotype of recombinant vEP84Ri correlates with the inducer-dependent expression of pEP84R, indicating that it is essential for the generation of infectious virus.

### Protein pEP84R is required for core assembly

**Fig 2:**
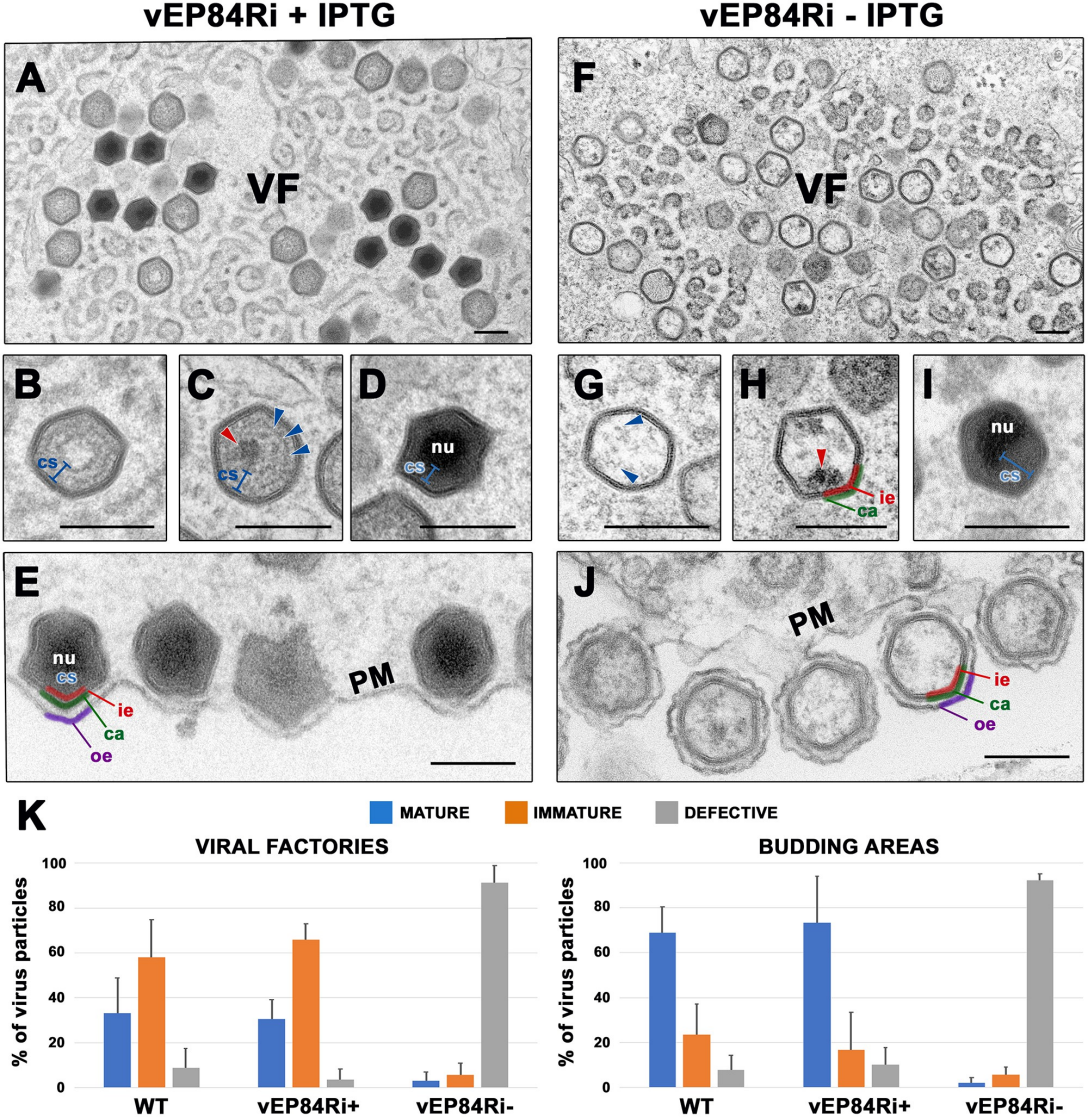
Protein pEP84R is required for core assembly. EM analysis of Vero cells infected with vEP84Ri virus for 20 h in the presence **(A-E)** or in the absence **(F-J)** of IPTG. Note that while under permissive conditions (+IPTG), the viral factories (VF) contain significant amounts of mature particles **(A)**, under non-permissive conditions (-IPTG), they contain essentially icosahedral empty particles **(F)**. Thus, whereas control vEP84Ri^+^ intermediates **(B, C)** contain a well-organized core shell (cs), consisting of a ~30-nm thick domain divided by a thin electron-dense layer (blue arrowheads in **C**), and sometimes also a centered developing nucleoid (nu, red arrowhead in **C**), the core region of vEP84Ri^-^ particles appear mostly empty, containing disorganized particulate material (blue arrowheads in **G**) and eventually an electron-dense incipient nucleoid (red arrowhead in **H**) attached to the inner envelope (ie, red). A minor population of the defective icosahedral viruses contained an acentric dense and large nucleoid surrounded by an asymmetric core shell **(I)**. Note also that vEP84Ri^-^ particles contain a normal outer capsid (ca, green) and acquire their outer envelope (oe, purple) by budding **(J)** at PM, as occurs with vEP84Ri^+^ particles **(E)**. Bars, 200 nm. **(K)** Quantification of mature, immature and defective vEP84Ri particles detected at 20 hpi under permissive (+) and non-permissive (-) conditions. The percentage of each virus category for each condition was estimated both in virus factories and budding areas. As a reference, wild type infections were also analyzed.

Next, we investigated the effect of blocking pEP84R expression on virus morphogenesis by EM analysis of cells infected with vEP84Ri under permissive or non-permissive conditions. In the presence of inducer, the appearance of the cytoplasmic virus factories (Fig 2A) was similar to that described for parental BA71V virus [9]. Thus, all stages of virus assembly, including precursor viral membranes, immature icosahedral particles (with a distinct, well-organized core shell but without nucleoid or with incipient genome condensation) (Fig 2B and 2C), and ´full`nucleoid-containing icosahedral mature particles (Fig 2D) were detected. In contrast, when the inducer was omitted, large quantities of icosahedral particles lacking a properly assembled core were observed at the assembly sites while immature and mature particles were rarely detected (Fig 2F). When compared with the viral icosahedral intermediates (Fig 2B and 2C) and the mature virus particles (Fig 2D) assembled in the presence of IPTG, the particles detected in the absence of inducer lacked the characteristic core shell structure as well as the electron-dense centered nucleoid (Fig 2G–2I). Rather, their core region appeared mostly empty or occupied by dispersed particulate material (Fig 2G). A minor but significant proportion (~20%) of the defective particles contained small and highly electron-dense aggregates, suggestive of an incipient nucleoid (Fig 2H), or displayed a large and acentric nucleoid surrounded by an irregular core shell (Fig 2I). Interestingly, the absence of pEP84R did not apparently affect the assembly of the inner viral envelope and the outer capsid nor the acquisition of the outer envelope by budding at the PM (Fig 2J). A quantitative analysis of the different types of virus particles (immature, mature and defective) detected in viral factories (Fig 2K, **left panel**) and budding areas (Fig 2K, **right panel**) showed, as expected, a significant increase in the proportion of mature particles (from ~30 to ~70%) at the exit sites in control infections with wild type and vEP84Ri virus in the presence of IPTG. The proportion of apparently defective particles remained below 10% for both controls and examined areas. In contrast, more than 90% of the vEP84Ri particles produced in the absence of IPTG were defective in terms of core shell and nucleoid organization in both areas.

Finally, we performed EM analysis to confirm the ultrastructural phenotype of recombinant vEP84Ri in porcine macrophages, the primary target cells for ASFV infection. As illustrated in S4E–S4H Fig, large amounts of core-less icosahedral defective particles were observed at 18 hpi in the assembly sites and the budding areas of vEP84Ri-infected macrophages in the absence of inducer. As a control, immature particles with a well-defined core shell as well as full nucleoid-containing virions were detected under permissive conditions (S4A–S4D Fig). Altogether, these observations indicate that protein pEP84R is required for the assembly of the viral core.

### Defective vEP84Ri^-^ particles lack major core shell and nucleoid proteins

**Fig 3:**
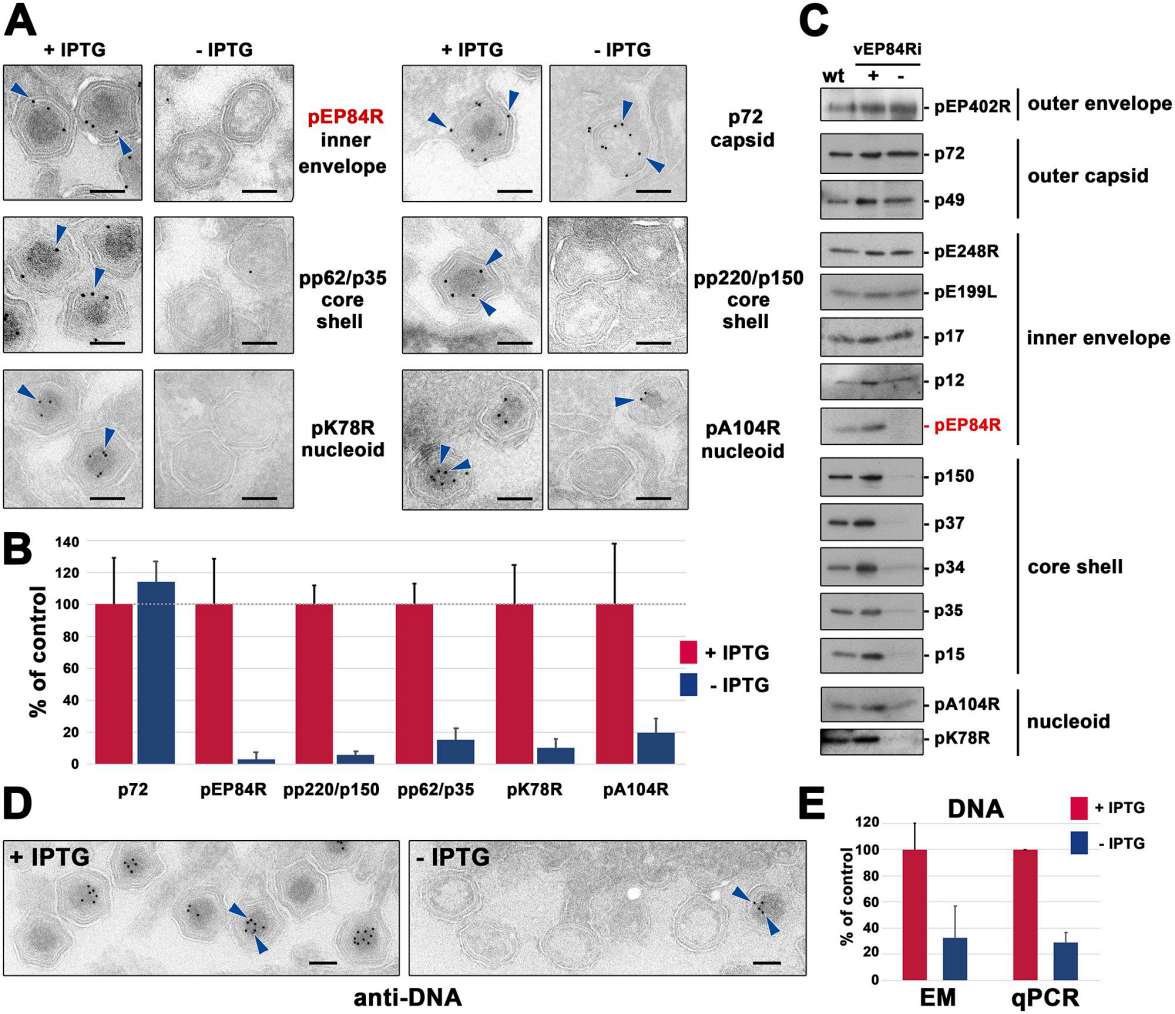
Defective vEP84Ri^-^ particles lack core shell and nucleoid components. **(A)** Immuno-EM of control (+IPTG) and defective (-IPTG) vEP84Ri particles at budding areas. Thawed cryosections of vEP84Ri-infected cells were incubated with antibodies against the inner envelope protein pEP84R, the outer capsid protein p72, the core shell proteins pp220/p150 and pp62/p35, and the nucleoid, DNA-binding proteins pK78R and pA104R, followed by protein A-gold (10 nm). The arrowheads indicate representative labeling. Bars, 100 nm. **(B)** Quantification of immunogold labeling. The number of gold particles per icosahedral virus detected under permissive (+IPTG) and non-permissive (-IPTG) conditions was quantified from 5–10 different budding areas. Data are expressed as a percentage (± SD) of the mean labeling density detected in control (+IPTG) condition **(C)** Protein composition of defective vEP84Ri particles. Equivalent amounts of purified extracellular control vEP84Ri^+^ and defective vEP84Ri^-^ particles were analyzed by Western immunoblotting for representative virion proteins localized at different layers. As a control, wild type (wt) ASFV particles were also analyzed. **(D)** DNA labeling of vEP84Ri particles. Cryosections of vEP84Ri-infected cells in the presence or absence of IPTG were incubated with anti-DNA mAb, a bridging rabbit anti-mouse antibody and protein A-gold (10 nm). The arrowheads indicate representative nucleoid labeling of control mature particles (+IPTG) as well as a small fraction of defective full particles (-IPTG) at budding areas. Bars, 100 nm. **(E)** DNA content of vEP84Ri particles. Left (EM): DNA immunolabeling of control (+IPTG) and defective (-IPTG) particles was quantified at different budding areas (>100 particles per condition). Data are expressed as a percentage (± SD) of positive labeling (>1 gold particle/virus particle) detected in control vEP84Ri (+IPTG) viruses. Right (qPCR): Percoll-purified vEP84Ri^+^ and vEP84Ri^-^ particles were subjected to DNA extraction and qPCR with a set of primers specific for ASFV gene *B646*. The amount of purified particles used was normalized for MCP content by western blot. Data are expressed as a percentage (± SD) of the DNA content detected in control vEP84Ri (+IPTG) particles.

To ascertain how the lack of pEP84R affects the core assembly at the molecular level, immunogold labeling with a set of anti-ASFV antibodies was performed on ultrathin sections of vEP84Ri-infected Vero cells in the presence or absence of IPTG. As illustrated in Fig 3A and quantified in Fig 3B, defective vEP84Ri particles produced under non-permissive conditions contained greatly reduced levels of pE84R as well as the core shell components pp220/p150 and pp62/p35. Also, the labeling of two major nucleoid proteins, the DNA-binding proteins pK78R and pA104R, was significantly diminished, with the exception of the minor fraction of defective particles containing an aberrant nucleoid. As a positive control, the signal detected for MCP p72 was similar under permissive and restrictive conditions.

In a complementary approach, highly purified extracellular defective vEP84Ri^-^ particles were analyzed by Western blot using antibodies against representative markers of the different virus layers. As a control, equivalent amounts of wild type and vEP84Ri viral particles produced under permissive conditions were also analyzed. As shown in Fig 3C, the defective vEP84Ri^-^ particles contained essentially no protein pEP84R as well as substantially reduced levels of major core shell and nucleoid proteins, while the amounts of the outer envelope, outer capsid and inner envelope markers were not significantly affected.

### DNA encapsidation is severely impaired in defective vEP84Ri^-^ particles

Next, the presence of the viral genome in defective vEP84Ri^-^ particles was analyzed. In a first approach, we performed immunogold labeling with an anti-DNA antibody to quantify the percentage of control (+ IPTG) and defective (-IPTG) particles labeled at the budding areas (Fig 3D). As shown in Fig 3E **(EM)**, the proportion of DNA+ labeled particles was about 3 times lower under restrictive than under permissive conditions. It is worth mentioning that a significant proportion of the nucleoid-containing particles were not labeled (Fig 3D, **+ IPTG**), possibly due to the limited accessibility of the anti-DNA antibody to the highly packaged viral genome.

In a complementary approach, equivalent amounts of purified vEP84Ri^+^ and vEP84Ri^-^ particles were subjected to DNA extraction and ASFV-specific qPCR. As shown in Fig 3E **(qPCR)**, the DNA content of vEP84Ri^-^ particles was about 4 times lower than that detected in control vEP84Ri^+^ virions, confirming that recombinant vEP84Ri displays a severe DNA packaging defect under restrictive conditions.

In conclusion, immunocytochemical and biochemical approaches indicate that pEP84R repression blocks the recruitment and/or the assembly of the core shell and nucleoid components, including the viral genome, into the virus particles.

### Protein pEP84R is required for the correct membrane targeting and proteolytic processing of core shell polyproteins

The core-less phenotype of defective vEP84Ri virus resembles that reported for ASFV recombinants that lack either pp220 or pp62 polyproteins [29, 30]. Therefore, we next explored the expression, proteolytic processing and subcellular localization of these precursors in vEP84Ri-infected cells under restrictive conditions. As shown in S5A Fig, the proteolytic processing of pp220 and pp62 was severely impaired in the absence of pEP84R expression. This was not due to the absence of the viral protease pS273R, as its expression levels were similar to those of control wt and vEP84Ri (+IPTG) infections.

**Fig 4:**
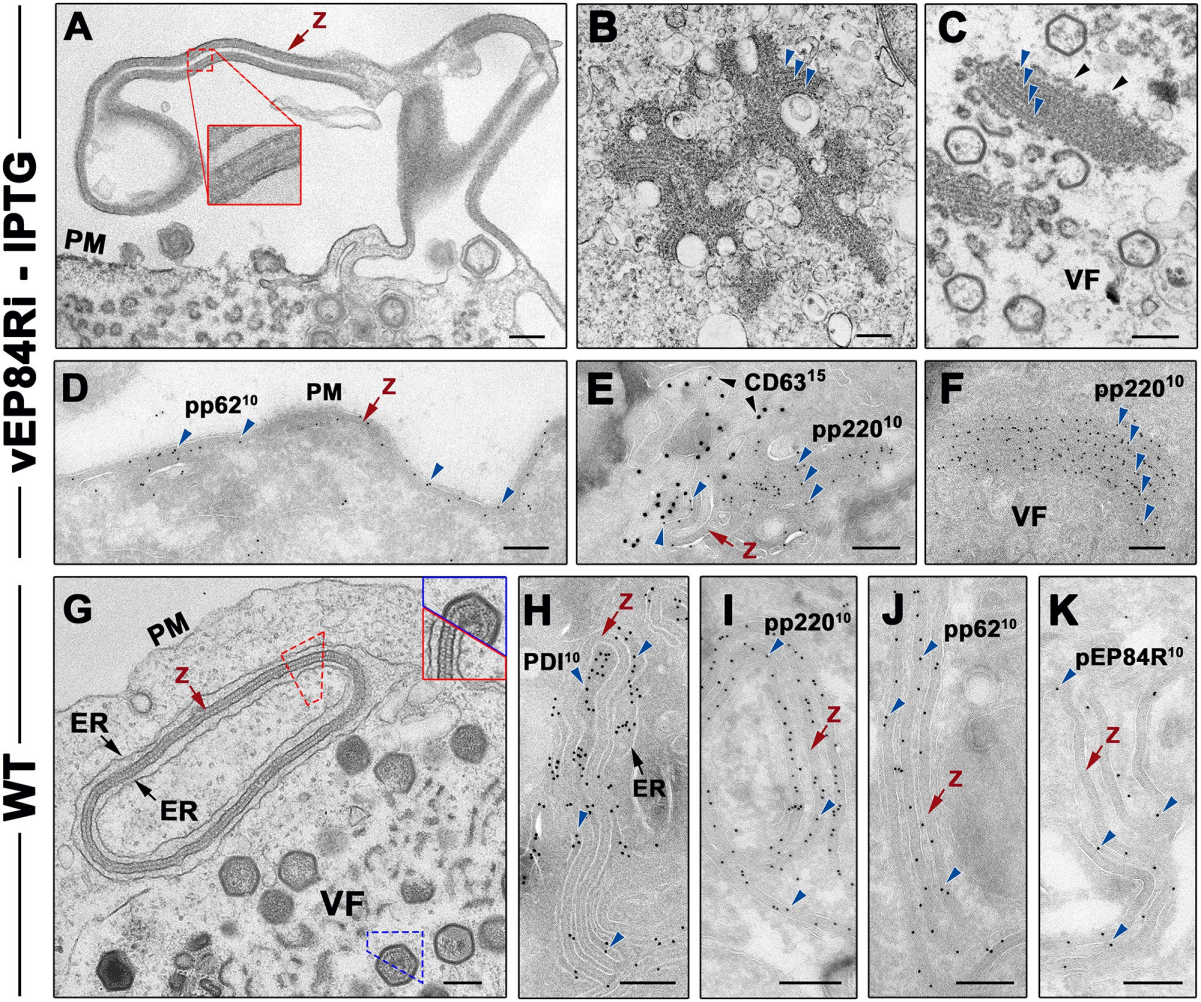
The absence of pEP84R leads to the accumulation of mistargeted core shell-like assemblies of pp220 and pp62 polyproteins. Conventional EM analysis (A-C and G) and immuno-EM (D-F and H-K) of cells infected with vEP84Ri in the absence of IPTG (A-F) or with wild type (WT) virus (G-K). In the absence of pEP84R, core shell-like assemblies of unprocessed pp220 and pp62 precursors, termed zippers (z), attach to the cytosolic face of the PM (A) and endosomal-lysosomal membranes (B). The inset in A shows a detail of a zipper associated to the PM. (C) Within the viral factories (VF), parallel stacks of core shell-like assemblies (blue arrows) displaying loose contacts to viral membranes (black arrowheads) are detected. Immunogold labeling of zippers with antibodies to pp62 (D) and pp220 (F) followed by protein A-gold (10 nm). (E) Double labeling of endosome-associated zippers with anti-pp220 and anti-CD63 antibodies followed by 10 and 15-nm gold conjugates, respectively. (G) ER-associated zipper (z) in close proximity to a wild type virus factory. The top right inset shows the similarity between the core shell of an assembling particle (blue outline) and a zipper structure (red outline). (H-K) Immuno-EM of ER-associated zippers with antibodies to luminal ER marker PDI (H), pp220 (I), pp62 (J) and pEP84R (K). Bars, 200 nm.

Next, we analyzed the distribution of pp220 precursor in vEP84Ri-infected cells by immunofluorescence. As shown in S5B Fig, while pp220 colocalized with MCP p72 at the virus factories under permissive conditions, a significant proportion of pp220 signal was detected in cytoplasmic inclusions outside the assembly sites in the absence of pEP84R. At the ultrastructural level (Fig 4A–4C), the absence of pEP84R led to the accumulation of core shell-like structures that appeared attached to the cytosolic side of the PM (Fig 4A) as well as endosomal and lysosomal-like compartments (Fig 4B). Within the viral factories, these structures could also be detected as parallel stacked arrays that occasionally bind to viral membranes through loose contacts (Fig 4C). These viral forms, previously described as zipper structures, represent aberrant assemblies of the unprocessed forms of polyproteins pp220 and pp62 [24]. Accordingly, all these categories of core shell-like structures were labeled with antibodies to pp220 and pp62 (Fig 4D–4F). Moreover, a significant proportion of them were associated to CD63+ vesicles (Fig 4E), which confirms their targeting to endosomal-lysosomal membranes besides the PM.

It is worth mentioning that zipper structures have been also detected at the periphery of the assembly sites of wild type virus infections [19] (Fig 4G), probably as a consequence of local excess of the core shell precursors. Remarkably, as opposed to the situation described for recombinant vEP84Ri, these zippers bind preferentially to ER cisternae, as confirmed by PDI immunolabeling (Fig 4G–4H). Moreover, immuno-EM showed that these ER-bound zippers contain not only the precursors pp220 (Fig 4I) and pp62 (Fig 4J) but also the transmembrane protein pEP84R (Fig 4K). Altogether, these results indicate that pEP84R is required for the correct targeting of the core shell precursors to the ER-derived inner viral envelope.

### Protein pEP84R guides the subcellular localization of core shell precursor pp220

**Fig 5:**
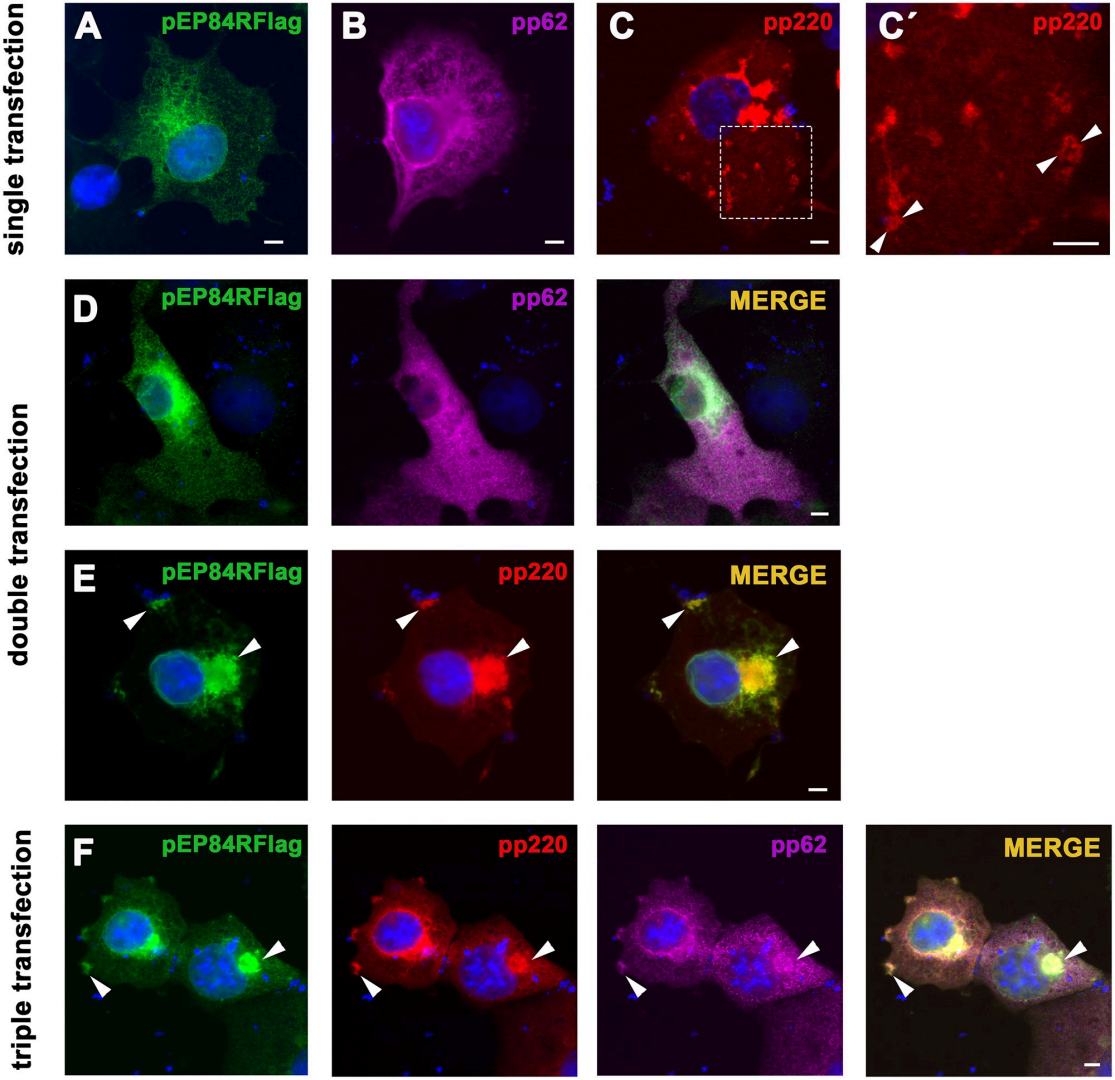
Protein pEP84R causes subcellular redistribution of polyprotein pp220. Transfected Cos cells expressing proteins pEP84R-Flag, pp62 and pp220 individually or in combination, were fixed and immunolabeled with rabbit antibodies to pEP84R **(A, D and F)** and pp220 **(E)**; mouse mAbs to pp62 **(B, D and F)**, pp220 **(C)** and Flag tag **(E)** or rat antibody to pp220 **(F)** as indicated. Note that pp220 **(C)** outlines scattered cytoplasmic compartments (inset **C´**) when expressed individually. Instead, when co-expressed with pEP84R (**E)**, both proteins colocalize to perinuclear areas and to a lesser extent to the cell surface (arrowheads). Note also the significant colocalization of pEP84R, pp220 and pp62 **(F)** at perinuclear areas and also at discrete PM patches (arrowheads). Bars, 10 μm.

**Fig 6:**
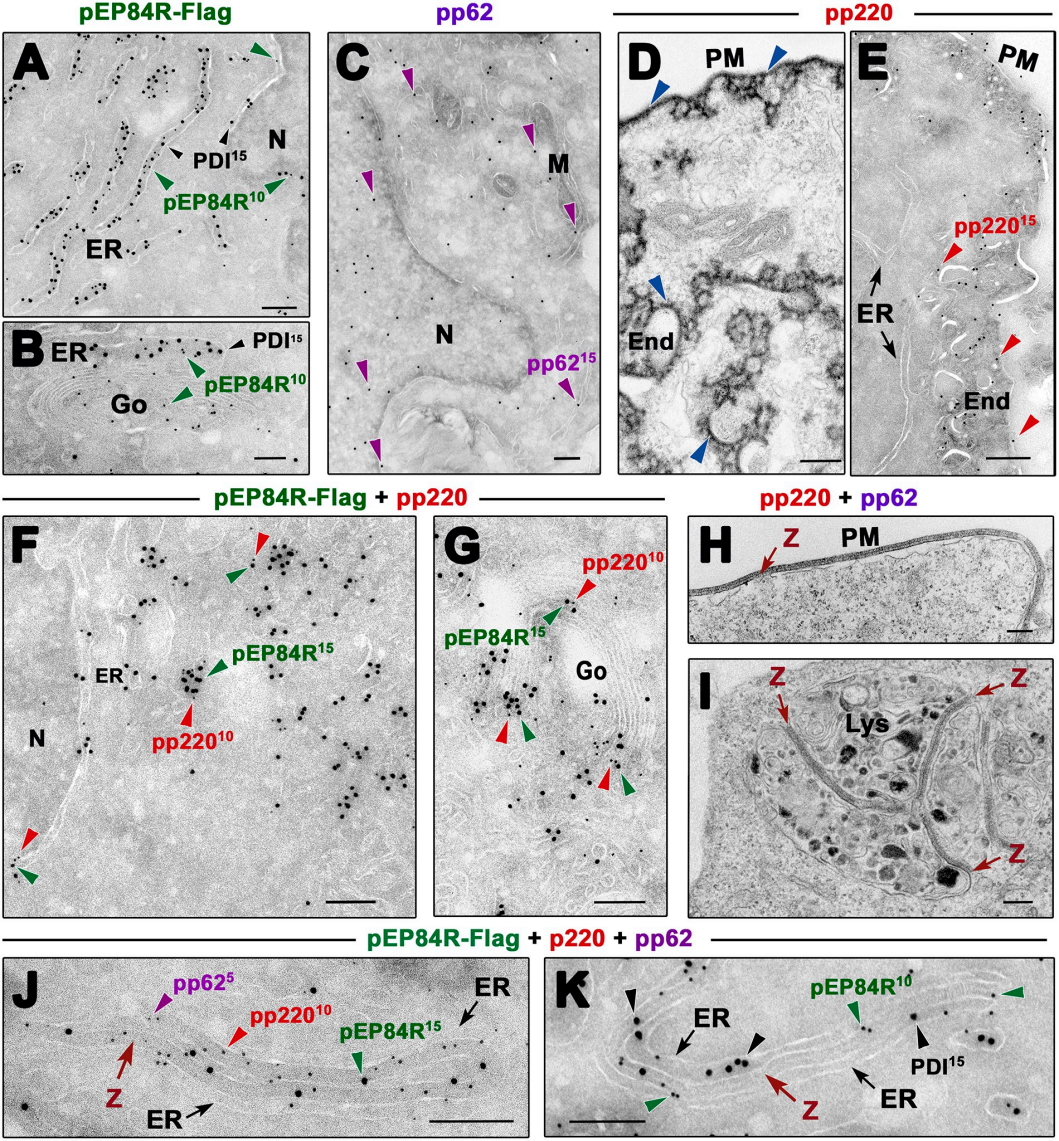
Protein pEP84R guides ER membrane targeting of pp220 and pp62 precursors. Transfected Cos cells expressing pEP84R-Flag, pp220 and pp62 individually or in combination, were fixed and processed for cryosectioning and immunogold labeling **(A, B, C, E, F, G, J and K)** or for conventional epoxy resin embedding **(D, E, H and I)**. **(A, B)** Double-labeling of pEP84R-Flag-expressing cells with rabbit anti-pEP84R (10-nm gold, green arrowheads) and mouse mAb anti-PDI (15 nm, black arrowheads) antibodies showing colocalization at ER **(A, B)**, including the nuclear envelope, as well as Golgi (Go) membranes **(B)**. **(C)** Immunostaining of pp62-expressing cells with a rabbit anti-pp62/p35 antibody (10-nm gold, purple arrowheads) reveals specific labeling at nucleus and cytoplasmic membranes. **(D)** Transfected polyprotein pp220 assembles into electron-dense coats (blue arrowheads) under the PM or surrounding cytoplasmic endosome (End) and lysosomes. **(E)** Immunolabeling of membrane-bound pp220 coats with an anti-pp220/p34 antibody (15-nm gold, red arrowheads). **(F-G)** Double labeling for pEP84R (10 nm, green arrowheads) and pp220 (15 nm, red arrowheads) in co-transfected cells. Note colocalization at the nuclear envelope **(F)**, Golgi membranes (Go) as well as perinuclear vesicles and cisternae **(F-G)** that probably belong to ER and Golgi networks. **(H-I)** Zipper (z) structures in cells co-expressing polyproteins pp220 and pp62. Note that these core shell-like structures are attached to the cytosolic face of the PM **(H)** as well as endosomal-lysosomal (Lys) membranes **(I)**. Triple immunogold labeling of ER-associated zippers with rabbit anti-pEP84R (15 nm, green arrowheads), rat anti-pp220 (10 nm, red arrowheads) and mouse anti-pp62 (5 nm, purple arrowheads) antibodies. **(J)** Double labeling with rabbit anti-pEP84R (10 nm, green arrowheads) and mouse anti-PDI (15 nm, black arrowheads) antibodies of ER-associated zippers (z) assembled in cells co-expressing pEP84R-Flag, pp200 and pp62. Bars, 200 nm.

To evaluate the hypothesis that pEP84R itself may guide the localization of the core shell precursors we performed immunofluorescence (Fig 5) and immuno-EM (Fig 6) approaches on transfected cells expressing proteins pEP84R-Flag, pp220 and pp62 individually or in combination. Expression of the target proteins was confirmed by Western immunoblotting (S6A Fig).

In keeping with the fluorescence pattern described before (Fig 5A, S2 and S7A Figs), immunogold labeling of pEP84R was primarily associated to the nuclear envelope and the Golgi complex along with membrane cisternae and vesicles that were identified as ER elements by pEP84R and PDI double-labeling (Fig 6A and 6B). Polyprotein pp62 was detected both in the nucleus and a variety of cytoplasmic compartments (ER, nuclear envelope, Golgi, mitochondria, PM) (Figs 5B and 6C S7B Fig), which suggests that it possesses an intrinsic capacity to bind lipid membranes. Subcellular fractionation of pp62-expressing cells confirmed such distribution and TX-114 phase separation showed that pp62 behaves as a peripheral membrane protein, as opposed to the transmembrane pEP84R-Flag used as a control (S6B Fig). Finally, transfected polyprotein pp220 accumulated in discrete cytoplasmic areas of varying sizes (Fig 5C and 5C´ and S7C Fig), some of them outlining intracellular compartments, with an overall pattern similar to that found in restrictive vEP84Ri infections (S5B Fig). Consistent with previous reports [29], immuno-EM analysis showed that those areas represent discrete patches of highly electron-dense coats underneath the PM or around endosomal-lysosomal compartments (Fig 6D and 6E).

Co-transfection experiments showed that the distribution pattern of pp62 was not significantly modified when co-expressed with pEP84R (Fig 5D and S7D Fig). In contrast, the subcellular localization of pp220 was drastically altered when co-expressed with pEP84R (Fig 5E, S7E and S7F Fig). Thus, most of pp220 largely redistributed to perinuclear areas, where it colocalized with pEP84R. Additionally, a small fraction of pEP84R colocalized with pp220 in peripheral inclusions close to the cell surface. At the EM level (Fig 6F and 6G), pEP84R and pp220 dual labeling showed their co-localization at the nuclear envelope, ER and Golgi membranes. In addition, perinuclear clusters of pEP84R-containing vesicles appeared embedded in an electron-dense matrix formed by pp220 precursor (Fig 6F). Overall, these results indicate that pEP84R guides membrane targeting of pp220, which strongly supports a direct interaction between them.

Since polyproteins pp220 and pp62 interact with each other to form zippers linked to the PM and endosomal-lysosomal compartments [24] (Fig 6H and 6I), we tested if both relocalize when co-expressed with pEP84R in transfected cells. As shown in Fig 5F and S7G–S7H Fig, proteins pEP84R, pp220 and pp62 colocalize at perinuclear areas and also in discrete cell surface fluorescent patches. EM analysis showed that co-expression of the three proteins led to the formation of membrane-bound zippers, as indicated by triple immunogold labeling for pEP84R, pp220 and pp62 (Fig 6J, S8A and S8B Fig). Remarkably, these zippers were mostly targeted to ER membranes, as demonstrated by double labeling with anti-pEP84R and anti-PDI antibodies (Fig 6K). Collectively, these findings strongly support the notion that pE84R itself mediates the membrane targeting of the core shell polyproteins pp220 and pp62 to the assembling viral particles during infection.

### Protein pEP84R interacts with the N-terminal region of polyprotein pp220

**Fig 7:**
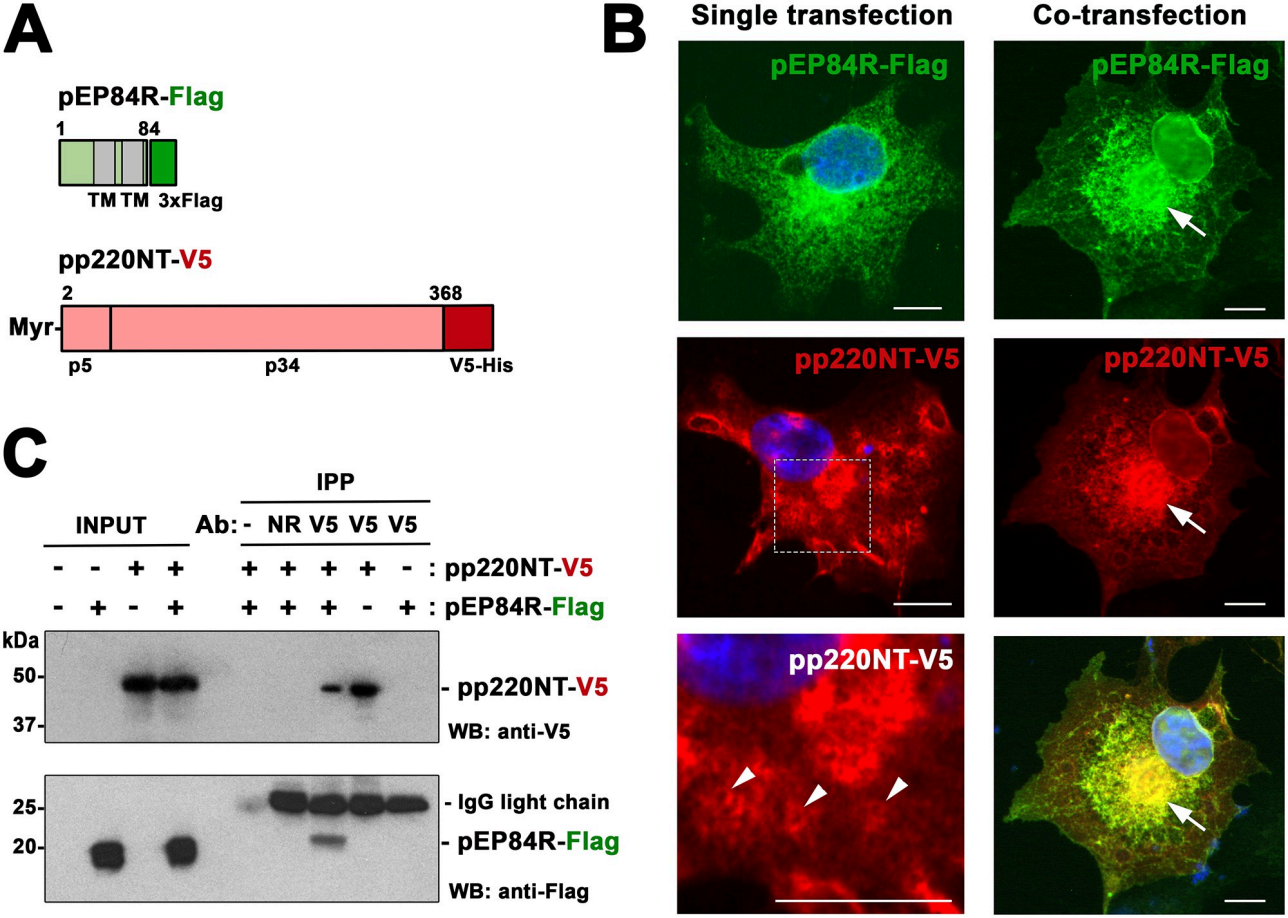
Transmembrane protein pEP84R interacts with the N-terminal region of polyprotein pp220. **(A)** Schematic representation of pEP84R-Flag and pp220NT-V5 constructs. The putative transmembrane segments (TM) of pEP84R, the N-myristoyl moiety of pp220NT, the His-V5 (pp220NT) and Flag (pEP84R) tags, and the regions corresponding to p5 and p34 products derived from pp220 are indicated. **(B)** Immunofluorescence detection of pEP84R-Flag and pp220NT-V5 in transfected cells. Cos cells transfected with pEP84R-Flag and pp220NT-V5 constructs individually or in combination, were immunolabeled with anti-pp220 (red) and anti-Flag (green) antibodies. Note vesicular-like pattern of pp220NT-V5 (middle left) indicated by arrowheads in a detail (bottom left) and colocalization (arrows in right panels) of pEP84R-Flag and pp220NT-V5 in co-transfected cells at the nuclear envelope and perinuclear areas. Bars, 10 μm. **(C)** Co-immunoprecipitation of pEP84R-Flag with pp220-NT-V5. Clarified lysates of Cos cells co-transfected with pEP84R-Flag and pp220NT-V5 constructs were incubated with no antibody (-), a non-relevant (NR) antibody against MCP p72 (mAb 1BC11) or an anti-V5 mAb followed by protein G magnetic beads. As negative controls, lysates of cells expressing pEP84R-Flag or pp220NT-V5 individually were immunoprecipitated with anti-V5 mAb. Total extracts (INPUT) and immunoprecipitated proteins (IPP) were analyzed by western blot with anti-V5 and anti-Flag mAbs. The molecular masses (in kDa) as well as the pEP84R-Flag and pp220NT-V5 bands are indicated.

Since membrane-anchoring of pp220 occurs through its N-terminal myristoyl group, we speculated that transmembrane pEP84R interacts with the N-terminal region of the polyprotein. To test this, we expressed an N-terminal fragment of pp220 comprising the p5 and p34 regions, fused to a V5-His tag (pp220NT-V5) (Fig 7A). As shown in Fig 7B and S9 Fig, immunofluorescence analysis showed that pp220NT-V5 preferentially displayed a cytoplasmic vesicular-like pattern made up of discrete, ring-shaped structures of heterogeneous size, as occurred with the full-length protein. However, when it was co-expressed together with pEP84R-Flag, it relocalized to perinuclear areas displaying a reticular-like pattern emerging from the nuclear envelope, where it was found together with pEP84R. This distribution resembles that of pEP84R when expressed individually, although with a more compact and perinuclear distribution.

To confirm the interaction of pEP84R with the N-terminal region of pp220, clarified lysates of cells transfected with both pEP84R-Flag and pp220NT-V5 constructs were immunoprecipitated with anti-V5 magnetic beads. Western blot analysis demonstrated that pEP84R-Flag co-immunoprecipitated with pp220NT-V5 (Fig 7C). No pEP84R-Flag band was detected when anti-V5 antibody was omitted or replaced by an irrelevant antibody, nor when anti-V5 antibody was incubated with cell lysates expressing only pEP84R-Flag.

In conclusion, the co-localization and co-immunoprecipitation of pEP84R and pp220NT in the absence of other viral proteins indicate a direct interaction between both polypeptides. This interaction provides a molecular mechanism to explain the crucial role of pEP84R in the membrane targeting of core assembly.

## Discussion

The unique architecture of the infectious ASFV particle, with two icosahedral protein shells and two lipoprotein membranes enclosing the genome-containing nucleoid [8, 11, 12], is the result of an intricate morphogenetic pathway that essentially takes place in perinuclear assembly sites and secondarily at the PM [16–18]. In the first location, the ER-derived inner viral envelope acts as a docking platform for the multiple interactions required for the assembly of the external and internal viral layers [9]. Previous studies have identified the major transmembrane protein p17 as responsible for the anchorage of the MCP p72 to the inner envelope, and hence for the assembly of the outer capsid [11, 12, 31]. The present report provides evidence for the pivotal role of the previously uncharacterized inner envelope protein, pEP84R, in the assembly of the viral core.

**Fig 8:**
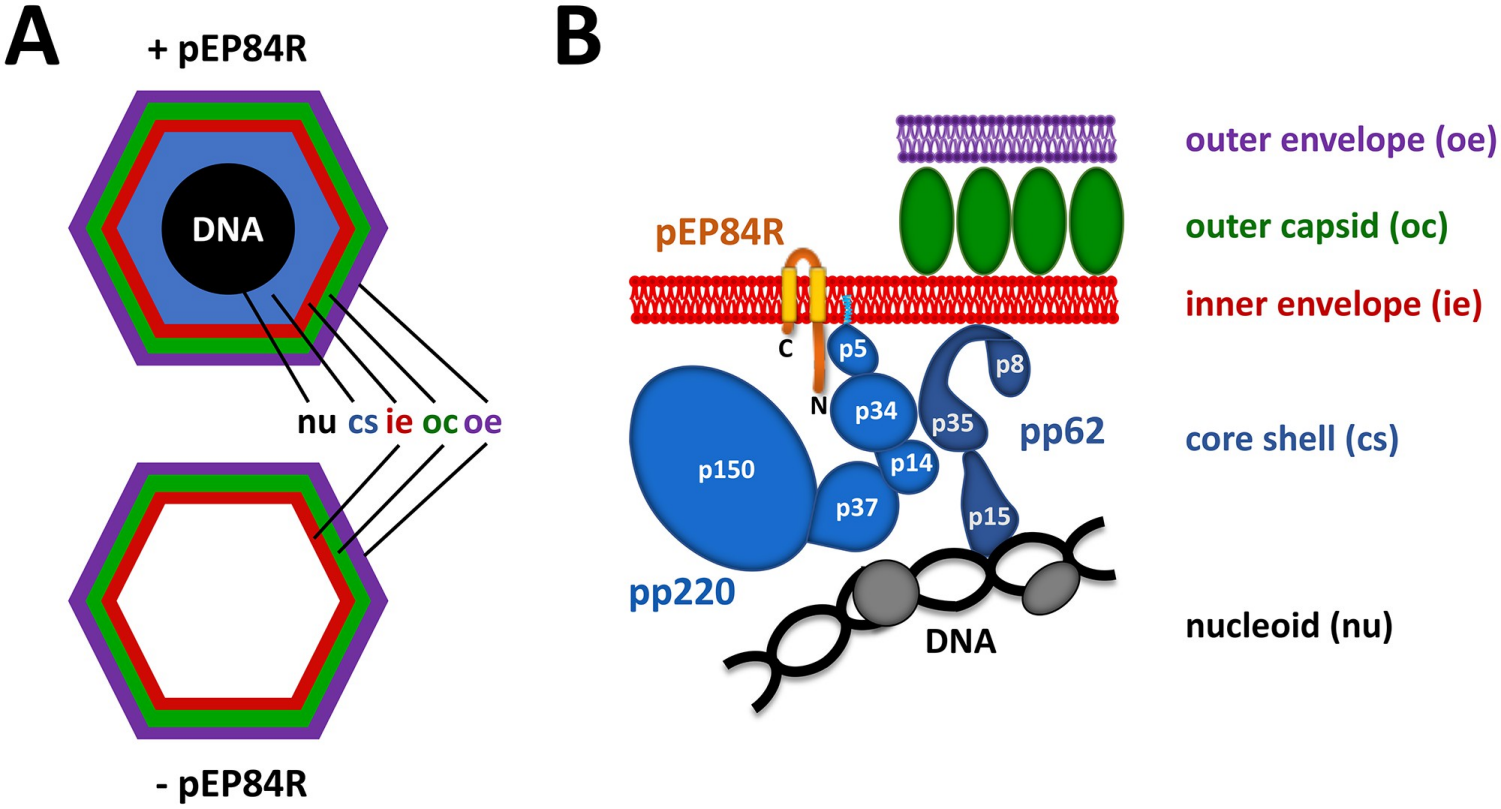
Role of pEP84R in ASFV core assembly. **(A)** Schematic depiction of the structure of ASFV particles generated in the presence (+) or absence (-) of pEP84R. The different virus layers are indicated. **(B)** Model for ASFV core assembly. Transmembrane protein pEP84R guides the assembly of the viral core beneath the inner envelope by interacting with polyprotein pp220, a binding that probably involves the N-terminal regions of both polypeptides. Precursor pp220, in turn, interacts with polyprotein pp62 to form the core shell, whose membrane anchoring would be further sustained by N-myristoylation of pp220 and a positively charged aa patch present in the p35 region of pp62. The interaction of the core shell with the underlying nucleoid likely involves the DNA binding domain of the pp62-derived product p15 together with other DNA-protein and protein-protein interactions not described so far. In the model, the names of the final processing products derived from pp220 (p5, p34, p14, p37 and p150) and pp62 (p15, p35 and p8) are indicated but not intended to represent actual structures or positions in the mature virion.

As shown above, the absence of pEP84R during ASFV infection leads to the formation of defective icosahedral particles lacking a proper core shell as well as the internal DNA-containing nucleoid. This core-less phenotype (Fig 8A) is remarkably similar to that reported for conditional lethal recombinants of polyproteins pp220 or pp62 [29, 30], which underlines a functional link between the inner envelope pEP84R and the core shell precursors. This connection is further supported by the observed mistargeting of both polyproteins, which formed core shell-like assemblies underneath the PM or around endosomal/lysosomal compartments.

As already mentioned, ASFV uses polyprotein expression, an uncommon gene expression strategy in DNA viruses [26], to synthesize the precursor polypeptides that form the core shell, a matrix structure that links the DNA-containing nucleoid to the inner envelope and hence to the outermost virus layers [9]. Similar to other well-known viral matrix proteins, such as the vesicular stomatitis virus M protein [32], the Ebola virus VP40 [33] or the influenza A virus M1 protein [34], ASFV core shell polyproteins show intrinsic capabilities of self-association and membrane anchoring. However, they require an extrinsic protein factor for proper membrane targeting. Thus, when expressed in the absence of other viral components, pp220 is able to multimerize to form electron-dense shells that bind to the cytosolic face of the PM as well as endocytic membranes [29]. Membrane anchoring of pp220 depends on the myristoylation of its N-terminal glycine residue, since a mutant non-myristoylated pp220 version does not associate to membranes but forms cytoplasmic aggregates [29]. Interestingly, when expressed together, pp220 and pp62 interact with each other to form core shell-like structures that, as also occurs in pp220-transfected cells or in restrictive vEP84Ri infections, bind mainly to the cell surface and endosomal-lysosomal membranes [24, 29], but not to the ER membranes that sustain ASFV assembly.

Our present results indicate that pEP84R provides a guiding cue for the correct membrane targeting of the core shell polyproteins. Besides the loss-of-function approaches, transfection experiments showed that pEP84R is able to recruit pp220 to perinuclear ER and Golgi membranes, where pEP84R preferentially targets. Moreover, pEP84R, pp220 and pp62 colocalize to form ER-bound core shell-like structures in transfected cells, as also observed in normal infections. Finally, the colocalization and coimmunoprecipitation of pEP84R with an N-terminal fragment of pp220 provides a mechanistic explanation to these findings.

On the basis of the previous evidence and the present results, we propose a model for ASFV core assembly (Fig 8B) in which transmembrane protein pEP84R guides the formation of the core shell beneath the inner viral membrane by interacting with polyprotein pp220. This interaction would involve the N-terminal regions of both polypeptides, as deduced from the co-immunoprecipitation experiments and their predicted membrane topology, although the possibility that other regions of pEP84R and pp220 are involved cannot be excluded. Thereafter, the multimerization of pp220 and its co-assembly with pp62 would give rise to an immature core shell, whose membrane anchoring might be further stabilized by the N-myristoyl group of pp220 and, perhaps, by other membrane binding signals present in the pp62 precursor or its mature products. In this connection, it has been shown that pp62-derived structural protein p35 binds *in vitro* to phosphatidylserine-enriched liposomes through a positively charged aa patch [35], which might also explain why pp62 behaves as a peripheral membrane protein when expressed in the absence of other viral components.

The reported defective phenotypes of inducible recombinants for pp220 and p62 along with that of pEP84R indicate that the assembly of the core shell is required for nucleoid formation [29, 30]. It is likely that this process involves specific interactions between some core shell components and the viral DNA and/or associated nucleoproteins. One possible link would be the pp62-derived product p15, which has been characterized as a disulfide-linked trimer that binds DNA *in vitro* [36, 37]. It should be noted, however, that polyprotein processing is not required for genome encapsidation [28], which suggests that DNA recruitment may precede the proteolytic maturation of the core shell.

At present, it is unknown what structural changes are triggered by polyprotein processing in the core structure although the recently identified internal icosahedral capsid that outlines the core shell of the mature virions [8, 12] seems to be a direct consequence of them. Thus, the resulting mature core could be considered as a *bona fide* nucleocapsid enwrapped by a lipid membrane (along with an extra outer capsid and outer membrane). In this context, ASFV core formation seems conceptually analogous in some aspects to that of retroviruses, in which the Gag polyprotein precursor acts as a primary driver for both the nucleocapsid assembly and its anchoring to the viral envelope [38–40]. Similar to most retroviral Gag precursors, pp220 uses N-myristoylation to facilitate membrane binding. Also, ASFV precursors contain not yet well-defined dedicated domains for membrane binding and targeting (N-terminal fragment of pp220 and p35), self-assembly and genome packaging and/or stabilization (p15), which in the Gag precursors have been clearly established (i.e. the matrix (MA), capsid (CA) and nucleocapsid (NC) domains among others). Finally, both viruses use polyprotein processing as a late maturational step required for infectious particle formation that involves significant structural rearrangements in the assembling particles [28, 40]. Gag processing gives rise to the distinct layers or substructures of the mature virion (i.e. MA, CA and NC) whereas that of ASFV core shell precursors would produce the internal capsid together with not yet elucidated innermost structural elements [8, 12]. Clearly, new functional as well as high-resolution structural studies on mature but also defective ASFV particles (especially those affected in core assembly) are needed to better understand the core organization and the molecular mechanisms underlying its proteolytic maturation.

At present, no sequence similarity has been found between pEP84R and other viral or cellular proteins. However, the closest relatives of ASFV, the amoeba-infecting faustovirus [41], pacmanvirus [42] and kaumoebavirus [43], together with the mollusk-infecting abalone asfa-like virus (AbALV) [44], encode homologs of polyproteins pp220 and pp62 as well as ASFV protease. Interestingly, the cryo-EM structure of faustovirus has unveiled an internal icosahedral capsid layer (besides the outer one) but not an interposed internal lipid membrane [45]. In contrast, the pacmanvirus cryo-EM structure contains an inner envelope enclosed by an external capsid, but not apparently an internal capsid similar to that of ASFV and faustovirus [42]. How these large and giant DNA viruses assemble and process their polyprotein precursors in likely distinct structural solutions is an intriguing question that awaits further functional and structural studies.

Currently, ASFV causes a worldwide animal health emergency that is aggravated by the absence of widely available vaccines or effective anti-viral strategies. In this context, the identification of a key orchestrator for core formation may be exploited for the development of antiviral compounds blocking its interaction with the core shell or for the generation of a safe new class of virus-like particle-based vaccines for the prevention of ASF.

## Materials and methods

### Cells and viruses

Vero and Cos-1 cells, derived from African green monkey kidney epithelia, were obtained from the American Type Culture Collection and grown at 37°C and 5% CO_2_ atmosphere in Dulbecco’s modified Eagle’s medium (DMEM) containing 5% fetal bovine serum (FBS), which was reduced to 2% during viral infection. Porcine alveolar macrophages (from a laboratory stock stored in liquid nitrogen) were cultured in DMEM supplemented with 10% heat-inactivated FBS. The Vero cell-adapted ASFV strain BA71V has been described before [46]. Unless otherwise specified, viral infections were carried out with extracellular particles collected from infection supernatants clarified by low-speed centrifugation (1,000 x g for 10 min).

### Antibodies

The affinity-purified rabbit antibody against the viral protein pEP84R was obtained from Davids Biotechnologie by immunization with the pEP84R-derived peptide ATEPEVGLPLLALQHSKS.

The following antibodies against ASFV polypeptides have been previously described [25–27, 31, 47–50]: rabbit polyclonal antibodies for p150, p37/p14 and p34, which are derived from polyprotein pp220 (pCP2475L), p15 and p35, derived from polyprotein pp62 (pCP530R), p72 (pB646L), p49 (pB438L), p17 (pD117L), p12 (pO61R), pE248R, pEP402R/CD2v, p32 (pCP204L), p10 (pK78R) and pA104R; rat serum to protein pE199L and mouse monoclonal antibodies (mAb) to proteins p150 (18H.H7 and 17A.H2) and p72 (19B.A2 and 17L.D3). The mouse mAb 17F.E10, previously described as an anti-p60/p37 antibody [50], has been re-characterized in the present study as a specific antibody to polyprotein pp62 (ORF CP530R) and its mature structural protein p35 by immunoblotting (S6 Fig), immunofluorescence (Fig 5B) and immuno-EM analyses (Fig 6C) of pp62-expressing Cos cells.

The sources of the mouse mAbs for host cell markers and tag epitopes were as follows: anti-β-Actin (clone AC-15, Sigma), anti-CD63 (H5C6, DSHB, University of Iowa), anti-PDI (1D3, Enzo Life Sciences), anti-ERGIC-53 (OTI1A8, Enzo Life Sciences); anti-GM130 (35/GM130, BD Transduction Laboratories), anti-DNA (IgM Ac 30–10, Sigma), anti-Flag (M2, Sigma), anti-V5 (clone V5-10, Sigma). The sheep anti-TGN46 (AHP500G) was from Bio-Rad and the rabbit anti-pan-AKT antibody (ab880) from Abcam.

### Expression plasmids

The plasmids pGEM-CP2475L and pGEM-CP530R, containing the pp220- and pp62-encoding genes, respectively, under control of the T7 polymerase promoter, have been described before [25, 27]. Plasmid pEP84R-Flag allows CMV promoter controlled expression of a C terminally 3x-Flag tagged pEP84R protein in eukaryotic cells. This was obtained in two steps. First, an *EP84R*-containing PCR product amplified with oligonucleotides AH406 (5’- CTTTCAGGGTACCGAAGAAGGATCCGCCACCATGCCTTATTCAAGAGATATC) and AH407 (5’- CAGAATTCCTCCTTGGATCCTTAGAATTATCTTTATAAATAAGAAAAAAACC) using purified viral DNA as a template was recombined using InFusion reagents (Clontech) into Bam HI-digested plasmid pIREScOFP (Stratagene). To avoid interference of OFP expression, the fragment containing the IRES sequence and the OFP encoding sequence was deleted from the resulting plasmid using Q5 site directed mutagenesis (NEB) with oligonucleotides AH435 (5’-GAGCATGCATCTAGAGGG) and AH436 (5’- TATGCAGTCGTCGAGGAA) following the manufacturer´s indications.

Plasmid pCP2475L(NT)-V5His to express the pp220NT-V5 fragment was obtained by HiFi Assembly (New England Biolabs). The fragment from *CP2475L* encoding the N-terminal residues 1 to 368, corresponding to mature proteins p5 and p34, was PCR amplified from plasmid pGEM-CP2475L using Q5 polymerase (NEB) and oligonucleotides AH408 (5’-CACTAGTCCAGTGTGGTGGAATTCGCCACCATGGGTAACCGTGGATC) and AH438 (5’- GCTGGATATCTGCAGAATTCTCACCCCCCTTTTTGG) and assembled with Bam HI-digested plasmid pEF-V5His B (Invitrogen). The final plasmids were sequenced to confirm the absence of unwanted mutations.

### Cloning of the plasmid for generation of recombinant vEP84Ri virus

In a first step, an inducible cassette was subcloned into Not I/Pac I digested plasmid pUC19 using InFusion (Stratagene) recombination to allow streamlined subcloning. This cassette bears the following relevant elements: E. coli lacI repressor coding gene under the control of the *U104L* early/late viral promoter, a beta-glucuronidase gene under the control of the strong late viral p72.4 promoter and finally, the inducible p72 derived promoter p72I*. The inducible cassette was PCR amplified using high fidelity Q5 polymerase (New England Biolabs) and oligonucleotides AH339 (5’-CGGTACCCGGGGATCGCGGCCGCTCGACGGATTTTAATTAGATTTGTG) and AH340 (5’-CGACTCTAGAGGATCTTAATTAATTGTTATCCGCTCACAATTTATATAATG) and plasmid pE199Li [49] as a template. The absence of unwanted mutations in the resulting plasmid pRepgus72I was verified by conventional Sanger sequencing. Next, a plasmid bearing appropriate flanking sequences for recombination was designed and synthesised in vitro (Genecust). This contained a 5’ flanking region spanning nucleotide positions 52400 to 53197 from Ba71V and a 3’ flanking region corresponding to positions 53198 to 53900. A single Not I site present in the 5’ flanking region within ORF *EP1242L* was abrogated by introducing a conservative single nucleotide mutation for cloning purposes. The flanking regions are separated by a short nucleotide stretch containing unique Pac I and Not I restriction sites which allow subcloning of the mentioned inducible cassette placing the initiator codon of *EP84R* immediately downstream of the inducible promoter, yielding the final plasmid used for recombination, pEP84Ri.

### Generation of an ASFV recombinant virus for the inducible expression of *EP84R* gene

A scheme of the ASFV recombinant, vEP84Ri, in which the expression of the *EP84R* gene is under the control of the E coli lac operator/repressor system is shown in S3 Fig. Recombinant virus vEP84Ri was generated as described previously [48]. Briefly, Vero cells were transfected with plasmid pEP84Ri and infected with BA71V virus in the presence of 1mM isopropyl-ß-D-thiogalactopyranoside (IPTG). At 72 hpi, the cells were harvested and the recombinant virus vEP84Ri was isolated by sequential rounds of plaque purification in the presence of inducer. To confirm the genetic structure of vEP84Ri, the corresponding viral DNA was purified as previously described [49] and subjected to Next Generation Sequencing (NGS). A DNA library was generated using 500 ng of purified viral DNA with the DNA Prep Tagmentation (Illumina) kit and the Illumina DNA/RNA UD Indexes Set A(IDT) adapters, following the manufacturer’s instructions. The resulting average insert size obtained for these libraries was 580 bp and NGS was performed on a MiniSeq sequencing system (Illumina), using a 2 x 150 run with the MiniSeq Mid Output kit (300-cycle) (Illumina). Reads obtained were mapped to the expected vEP84Ri reference genome with Bowtie2 aligner software using default parameters. Average coverage obtained for a covered base was 541x and the corresponding sequence analysis discarded the existence of any additional and non-desirable mutations in vEP84Ri genome. Files containing raw reads and the corresponding alignment against the reference genome sequence were deposited at European Nucleotide Archive under project number PRJEB56760.

### Plaque assay

Vero cell monolayers, seeded in six-well plates, were infected with recombinant vEP84Ri, or parental BA71V as a control. After 1 h, the inoculum was removed and the cells were overlaid with DMEM containing 0.6% Noble agar and 2% FBS in the presence or absence of 1 mM IPTG. The medium was removed 5–7 days later after paraformaldehyde (PFA) fixation and the monolayers stained with 1% crystal violet.

### One-step virus growth curves

Vero cell monolayers, seeded in 24-well plates, were infected with 5 pfu/cell of recombinant vEP84Ri or parental BA71V. After a 1-h adsorption, the cells were incubated in medium supplemented with 2% FBS in the presence or absence of 1 mM IPTG. Finally, the infected cells were harvested together with their culture supernatants at different times post-infection, sonicated and titrated by plaque assay in the presence of 1 mM IPTG.

### Topology and structural predictions

Putative transmembrane regions in BA71V as annotated on the Uniprot website (https://www.uniprot.org/uniprot/Q07383) are considered. The possible protein topology was predicted using TMHMM 2.0 at https://services.healthtech.dtu.dk/service.php?TMHMM-2.0.

### Analysis of the membrane association of protein pEP84R

Mock- and ASFV-infected cells were fractionated at 20 hpi into nuclear, cytoplasmic and membrane fractions, according to the instructions of the Minute Plasma Membrane Protein Isolation and Cell Fractionation Kit (Invent Biotechnologies). For alkaline carbonate extraction, membrane fractions were treated with 0.1 M Na_2_CO_3_ pH 11.5, for 30 min at 4°C and centrifuged for 10 min at 16,000 x g. Both supernatant and sediment were dissociated with electrophoresis sample buffer (2% SDS, 100 mM DTT, 125 mM Tris-HCl, pH 6.8). For Triton X-114 treatment, the membrane fraction was resuspended in 2% Triton X-114, 150 mM NaCl, and 10 mM Tris-HCl pH 7.5, incubated for 10 min at 4°C and then transferred to 30°C for 20 min. After phase separation by centrifugation, the lower detergent-rich phase and the upper aqueous phase were dissociated as described above. Equivalent amounts of the resulting fractions were analyzed by Western immunoblotting.

### Purification and composition analysis of recombinant vEP84Ri particles

Extracellular vEP84Ri particles were purified from clarified infection supernatants by Percoll gradient sedimentation [51], with slight modifications. In brief, Vero cells seeded in 50 150-mm plates were infected with vEP84Ri virus (MOI > 3 pfu/cell) in the presence or absence of IPTG. At 36 hpi, the infection supernatants were collected and clarified at 1,000 x g for 10 min to remove cell debris. The extracellular particles were then sedimented in a GS3 Sorvall rotor at 12,000 x g for 6 h at 4°C. The virus pellets were resuspended in PBS, sonicated and mixed with Percoll to a final concentration of 45%, and subjected to equilibrium gradient sedimentation in a TLA-100.3 rotor (Beckman) for 30 min at 37,000 x g and 4°C. After gradient fractionation, aliquots were analyzed by Western immunoblotting with an anti-p72 antibody to identify the virus-containing fractions, which were pooled and stored at—70°C until use.

For the analysis of the vEP84Ri protein composition, equivalent amounts of Percoll-purified vEP84Ri^+^ and vEP84Ri^-^ particles, adjusted by their MCP p72 content, were analyzed by immunoblotting with specific antibodies to representative markers of the different virus layers. As a reference, Percoll-purified wild type virus (BA71V strain) was also analyzed.

For the analysis of the DNA content, triplicate samples containing 2 μg of purified vEP84Ri^+^ and vEP84Ri^-^ particles were mixed with 100 ng of purified genomic DNA from ectromelia virus (ECTV), which was used as spike-in control for normalization, and subjected to DNA extraction and purification with the GeneJET Whole Blood Genomic DNA Purification Mini Kit (Thermo). Purified DNAs (2 ng/well in triplicate) were analyzed by qPCR with specific primers for *B646L* gene from ASFV strain BA71V (forward: 5´-CCAAACAGCAGGTAAACAAGA and reverse: 5´-GAATGGATACCGAGGGAATAG) and ECTV *A10L* gene (forward: 5´-CGCAATGCCATACAACATCT and reverse: 5´-TCCAATGCTTGGCTGACTAA) in a CFX384 Real Time System C1000 Thermal Cycler (Bio-Rad) using SSoFast EvaGreen Supermix (Biorad) and the following PCR cycling profile: 3 min x 95°C + (5 sec x 95°C and 5 sec x 60°C) x 40 cycles. After PCR amplification, melting curve analysis was performed for each reaction to verify PCR specificity. Relative quantification (2^–ΔΔCq^) of ASFV DNA was performed with GenEx software (MultiD Analyses AB, Sweden) using spike-in control and MCP p72 content of the virus samples for normalization. Data are expressed as a percentage (± SD) of the normalized DNA content detected in control (vEP84Ri +IPTG) particles.

### Western blot analysis

Cells and purified virus samples were dissociated in Laemmli buffer, heated at 90°C for 5 min and electrophoresed on 12% SDS-polyacrylamide gels. Proteins were transferred to PVDF membranes (0.2 μm, Bio-Rad) and incubated overnight at 4°C with the primary antibodies and for 1 h at RT with anti-rabbit, anti-rat or anti-mouse secondary antibodies conjugated to horseradish peroxidase (GE Healthcare) or TidyBlot (Biorad). Blots were developed with ECL prime reagent (GE Healthcare) on conventional film autoradiography.and, when necessary, scanned and quantified with ImageStudioLite (Li-cor).

### Transfection experiments

For the transient expression of pEP84R-Flag, pp220, pp62 and pp220NT-V5 proteins, Cos cells were grown to 80% confluency in DMEM at 5% FBS onto 35-mm dishes or 7-mm coverslips in 48-well plates. Cells were infected with 5 pfu/cell of a recombinant vaccinia virus (VACV), encoding the bacteriophage T7 RNA polymerase, in DMEM at 2% FBS. After 1h of adsorption, the cells were washed with DMEM and transfected for 12–20 h with the corresponding DNA plasmids in which the target genes are under control of the T7 promoter. Transfections were performed with lipofectamine 3000 transfection reagent (Thermo) in Opti-Mem (Life Technologies) according to the manufacturer’s indications. To prevent VACV late expression and morphogenesis, the DNA replication inhibitor ara-C (50 μg/ml) was added to the media throughout the infection/transfection experiment. At the end of the transfection period, the transfected cells were fixed for immunofluorescence or EM analysis or dissociated for immunoprecipitation or immunoblotting, as indicated.

### Co-immunoprecipitation assays

Transfected Cos cells, plated at 80% confluency in 35 mm plates, were lysed in 1ml of 0.1% Triton X-100 in PBS containing a protease inhibitor cocktail (complete mini-EDTA free, Sigma). Cell extracts were vortexed and clarified by centrifugation at 16,000 x g for 10 min at 4°C. For immunoprecipitation assays, typically 200 μl of each supernatant were incubated O/N at 4°C under rotation with 5 μg of purified anti-V5 mAb (IgG1, Sigma), anti-Flag M2 mAb (IgG1, Sigma) or a non-relevant mAb (1BC11 anti-p72, IgG1 Ingenasa). Then, 40 μl of protein G-conjugated magnetic beads (Dynabeads, Invitrogen) were added and incubated for 5h at 4°C under rotation. Beads were washed five times with 0.1% Triton in PBS and bound proteins were eluted in 30 μl of 2X Laemmli buffer and boiled at 95°C for 3 min. Input extracts and immunoprecipitated samples were analyzed in 12% polyacrylamide SDS-PAGE followed by western blot with anti-V5 and anti-Flag mAbs.

### Immunofluorescence microscopy

ASFV-infected Vero cells or transfected Cos cells, seeded on glass coverslips, were infected with parental ASFV virus or recombinant vEP84Ri for the indicated times, washed with PBS and fixed with methanol at -20°C for 5 min or with 4% PFA in PBS for 15 min at RT. Aldehyde-fixed cells were permeabilized with 0.1% saponin or 0.1% TX-100 in PBS for 5 min at RT, quenched with 50 mM NH_4_Cl for 5 min and blocked with 10% FBS for 5 min. The permeabilized infected cells were incubated for 45 min at RT with primary antibodies and 30 min at RT with Alexa-labeled secondary antibodies (Thermo Fisher). Cell nuclei were labeled with Hoechst 33258 (5 μg/ml). Coverslips were mounted with ProLong Glass Antifade Mountant (ThermoFisher) on microscope slides. Images were recorded with a Leica DMI6000B automated inverted microscope equipped with a Hamamatsu Orca R2 digital camera or a Zeiss LSM880 confocal laser scanning microscope.

### Transmission electron microscopy

ASFV-infected Vero cells and macrophages as well as transfected Cos cells were fixed with 4% PFA and 2% glutaraldehyde (GA) in 0.1 M phosphate buffer (PB, pH 7.4) for 120 min at RT. Post-fixation was carried out with 1% OsO_4_ and 0.8% K_3_Fe(CN)_6_ in water at 4°C for 1 h. Samples were dehydrated with ethanol and embedded in Epoxy, TAAB 812 Resin (TAAB Laboratories) according to standard procedures. After polymerization, ultrathin sections of about 90 nm were obtained and stained with uranyl acetate and lead citrate according to standard procedures. Samples were examined in a Jeol JEM-1010 electron or a Jeol 1400Flash microscope operating at 80 and 100 kV, respectively. Images were recorded with a TemCam-F416 (TVIPS, Germany) or a OneView (Gatan, USA) CMOS digital camera.

For virus quantification, a number of 240–260 (virus factories) or 100–120 (budding areas) closed icosahedral virus profiles per condition were counted at a magnification of 10,000–20,000X from 5–10 different areas. Virus particles containing a well-organized core shell and a centered nucleoid of normal size were classified as mature viruses. Particles displaying a well-organized core shell but lacking a centered normal nucleoid were considered as immature. Virus particles lacking a well-organized core shell and a centered normal-sized nucleoid were classified as defective. Data were expressed as the mean percentage (+/- SD) of each virus category per condition.

For immunoelectron microscopy, infected or transfected cells were *in situ* fixed for 2 h at RT with 4% PFA or 2% PFA and 0.2% GA in 0.1 M PB. Then, cells were embedded in 10% (w/v) gelatin, cryoprotected overnight in cold sucrose 2.3 M and rapidly frozen in liquid nitrogen. Ultrathin (90-nm) cryosections were obtained with a Leica EM FCS6 cryo-ultramicrotome at -120°C and retrieved with a 1:1 mixture of sucrose 2.3 M and 2.0% methylcellulose. Immunogold labeling was performed essentially as described [52] with slight modifications. For single immunolabeling with rabbit anti-ASFV antibodies, thawed cryosections were incubated with the primary antibody for 40 min at RT followed by protein A (PA) conjugated to 10 (PAG10) or 15-nm gold particles (PAG15) (EM Laboratory, Utrecht University) for 30 min at RT. Labeling with rat anti-pp220/p150 antibody was performed with goat anti-rat antibody conjugated to 10-nm gold particles (British Biocell, UK). For mouse mAbs for DNA, CD63 and PDI, the signal was amplified with bridging rabbit anti-mouse (RAM) IgG (Dako) followed by PAG10 or PAG15. Primary antibodies and gold conjugates were diluted in PBS containing 5% FBS. Sections were stained with a mix of 1.8% methylcellulose and 0.4% uranyl acetate before visualization.

For immuno-EM quantification with anti-ASFV antibodies, the mean labeling density (gold particles/virus) was obtained from 5–10 different budding areas (collectively containing from 60 to 130 closed virus profiles) for each antibody and condition (vEP84Ri +/- IPTG). For comparison, the mean labeling densities of vEP84Ri^-^ particles were expressed as a percentage (+/- SD) of those detected in control (vEP84Ri^+^) particles. For quantification of DNA labeling, the percentage of virus particles with at least 1 gold particle was estimated in 6–7 different budding areas. Data were expressed as the mean percentage (+/- SD) of DNA+ particles relative to control (vEP84Ri^+^) particles. Double and triple labelling procedures were set up for each combination and performed following protocols from the Electron Microscopy facility (EMF) at CBMSO. Details are available upon request.

### Statistical analysis

Unless otherwise indicated, the data are representative of at least three independent experiments, and values are given as the mean of triplicates ± standard deviation (SD).

## Supporting information

S1 Data Excel spreadsheet containing, in separate sheets, the underlying numerical data and statistical analysis for Figs 1H, 2K and 3B and 3E. (XLSX)

S1 Fig Multiple sequence alignment of pEP84R from 17 different ASFV strains. The aa sequences of pEP84R orthologues from different ASFV strains were retrieved from Genbank at the NCBI (https://www.ncbi.nlm.nih.gov/) and the corresponding accession numbers for each are: BA71V (NP_042748.1); Ken06.Bus (YP_009702953.1); Georgia_2007 (YP_009927178.1); China_2018(AYW34026.1); NHV (YP_009702621.1); OURT 88/3 (YP_009703662.1); Malawi LIL 20 (P0CAL5.1); Pr4 (P0CAL6.1); Namibia_Wart80 (P0CAL7.1); ASFVK49 (QZK26757.1); Liv13/33 (QID21215.1); Zaire 1977 (QII88576.1); RSA_2_2008 (QGM12834.2); KEN-50 (P0CAL4.1); Uvira (QRY19081.1); Ken05/Tk1 (YP_009702788.1); Ken.rie1 (CAD7112270.1). The sequences were aligned using Clustal Omega software at the EMBL-EBI (https://www.ebi.ac.uk/Tools/msa/clustalo/). Identical aa residues in all aligned sequences are designated with an asterisk at the bottom. Putative transmembrane regions in BA71V as annotated on the Uniprot website (https://www.uniprot.org/uniprot/Q07383) are indicated. The possible topology as predicted by TMHMM 2.0 at (https://services.healthtech.dtu.dk/service.php?TMHMM-2.0) is shown above the sequence, with “in” indicating the cytoplasmic side of the membrane.
(TIF)

S2 Fig Subcellular localization of pEP84R in transfected cells. Cos cells were transfected with a plasmid containing *EP84R* gene fused to a C-terminal 3XFlag-tag epitope sequence (pEP84R-Flag). At 20 h, cells were fixed and immunolabelled with rabbit anti-pEP84R **(A, B, C)**, or mAb anti-Flag **(D)**, and antibodies against ER (PDI; **A**), ERGIC (ERGIC-53; **B**), cis-Golgi (GM130; **C**) and TGN (TGN-46; **D**) markers. Note the partial colocalization of pEP84R-Flag with PDI at the nuclear envelope and peripheral ER, and with ERGIC, Golgi complex and TGN markers at perinuclear areas. Bars, 10 μm.
(TIF)

S3 Fig Genomic structure of inducible virus vEP84Ri. The recombinant virus was obtained by homologous recombination of parental ASFV genome (wt) with an inducible cassette containing a late, IPTG-dependent strong promoter (p72I*) for *EP84R* gene expression, a copy of E. coli lac repressor gene (*lacI*) and a reporter gene (*gusA*) used for selection and purification of the recombinant virus. ASFV genes in the proximity of the recombination area as well as the left and right flanking regions are indicated.
(TIF)

S4 Fig EM analysis of vEP84Ri-infected swine macrophages. Porcine alveolar macrophages were infected with vEP84Ri for 18h in the presence **(A-D)** or in the absence **(E-H)** of IPTG. Panels **B** and **F** show higher magnification images of the viral factory areas delimited in **A** and **E**, respectively. Note that while under permissive conditions (+IPTG), the viral factories (VF) contain significant amounts of immature particles with a well-organized core shell (arrowheads in **B**) and mature virions, under non-permissive conditions (-IPTG), they contain essentially core-less particles (**F**). Note also that defective vEP84Ri- particles exit by budding from the PM (**G-H**), as occurs with ´full`vEP84Ri+ particles (**C-D**). The different virus layers (nucleoid (nu), core shell (cs), inner envelope (ie), capsid (ca) and outer envelope (oe)) of budding vEP84Ri+ (inset **D**) and vEP84Ri- (inset **H**) particles are indicated. Nucleus (N), plasma membrane (PM). Bars, 1 μm (A, E) and 200 nm (B-D and F-H).
(TIF)

S5 Fig Expression of pEP84R is required for proteolytic processing and proper localization of ASFV polyproteins. **(A)** Extracts of Vero cells mock-infected (Mock) or infected with parental BA71V (wt) or recombinant vEP84Ri viruses in the presence (+I) or absence (-I) of IPTG were analyzed by immunoblotting with antibodies against the mature products derived from polyprotein pp220 (upper) and pp62 (middle) and protease pS273R (bottom). As a control, purified ASFV particles (V) were also analyzed. Note that polyprotein processing is strongly impaired under restrictive conditions. The positions of polyprotein pp220 and pp62, the intermediate processing products pp90 and pp46, the mature products p150, p37, p34, p35 and p15, and the viral protease pS273R are indicated. Molecular masses are indicated on the left. **(B)** Immunofluorescence labeling of pp220 (green) and MCP p72 (red) in vEP84Ri-infected cells in the presence (+) or absence (-) of IPTG. Nuclear and viral DNA (blue) was stained with Hoechst 33258. The arrows indicate virus factories (VF) whereas the arrowheads indicate cytoplasmic pp220 accumulations outside the virus assembly sites under non-permissive conditions. Bars, 5 μm.
(TIF)

S6 Fig Transient expression of pEP84R-Flag, pp220 and pp62 and membrane association of pEP84R-Flag and pp62. **(A)** Transfected Cos cells expressing proteins pEP84R-Flag, pp62 and pp220, individually or in combination, were analyzed by immunoblotting with antibodies to the target proteins. **(B)** Membrane-association of pEP84R-Flag and pp62. Transfected Cos cells expressing proteins pEP84R-Flag or pp62 were fractionated into cytosolic (C) and membrane/particulate (M) fractions. Also, the membrane fractions were subjected to TX-114 phase separation to obtain aqueous (A) and detergent-rich (D) phases. Equivalent fractions were analyzed by immunoblotting. Note that while pEP84R-Flag behaves as an integral membrane protein, pp62 behaves as a peripheral membrane protein. Molecular masses (in kDa) and pp220, pp62 and pEP84R-Flag bands are indicated.
(TIF)

S7 Fig Protein pEP84R causes subcellular redistribution of polyprotein pp220. Transfected Cos cells expressing proteins pEP84R-Flag, pp62 and pp220 individually or in combination, were fixed and immunolabeled with rabbit antibodies to pEP84R **(D, E, G and H),** pp62 **(C)** and pp220 **(B and F)**; mouse mAbs to pp62 **(D, G and H)**, pp220 **(E)** and Flag tag **(A and F)** or rat antibody to pp220 **(G and H)** as indicated. Note that pp220 and pEP84R colocalize **(E and F)** to perinuclear areas and to a lesser extent to the cell surface (arrowheads). Note also the significant colocalization of pEP84R, pp220 and pp62 **(G and H)** at perinuclear areas (arrowheads). Bars, 10 μm.
(TIF)

S8 Fig Co-expression of pEP84R and ASFV polyproteins produces ER-associated core shell-like structures. Transfected Cos cells co-expressing pEP84R-Flag, pp220 and pp62, were processed for conventional epoxy resin embedding **(A)** or cryosectioning and immunogold labeling **(B)** for pEP84R (15 nm, green arrowhead), pp220 (10 nm, red arrowhead) and pp62 (5 nm, purple arrowhead). Note the formation of zipper (z) structures associated to ER cisternae **(A)**, which are immunolabeled for the three viral proteins **(B**). Bars, 200 nm.
(TIF)

S9 Fig Immunofluorescence detection of pEP84R-Flag and pp220NT-V5 in transfected cells. Cos cells transfected with pEP84R-Flag and pp220NT-V5 constructs individually or in combination, were immunolabeled with anti-pEP84R (green) and anti-V5 (red) antibodies. Note the vesicular-like pattern of pp220NT-V5 (middle left) indicated by arrowheads in a detail (bottom left) and colocalization (arrows in right panels) of pEP84R-Flag and pp220NT-V5 in co-transfected cells at perinuclear areas. Bars, 10 μm.
(TIF)
