# Supplementary figures and images for: African swine fever virus transmembrane protein pEP84R guides core assembly
Source: PLoS Pathog. 2023 Jan 30;19(1):e1011136. doi: 10.1371/journal.ppat.1011136 (PMC9910796; doi:10.1371/journal.ppat.1011136)

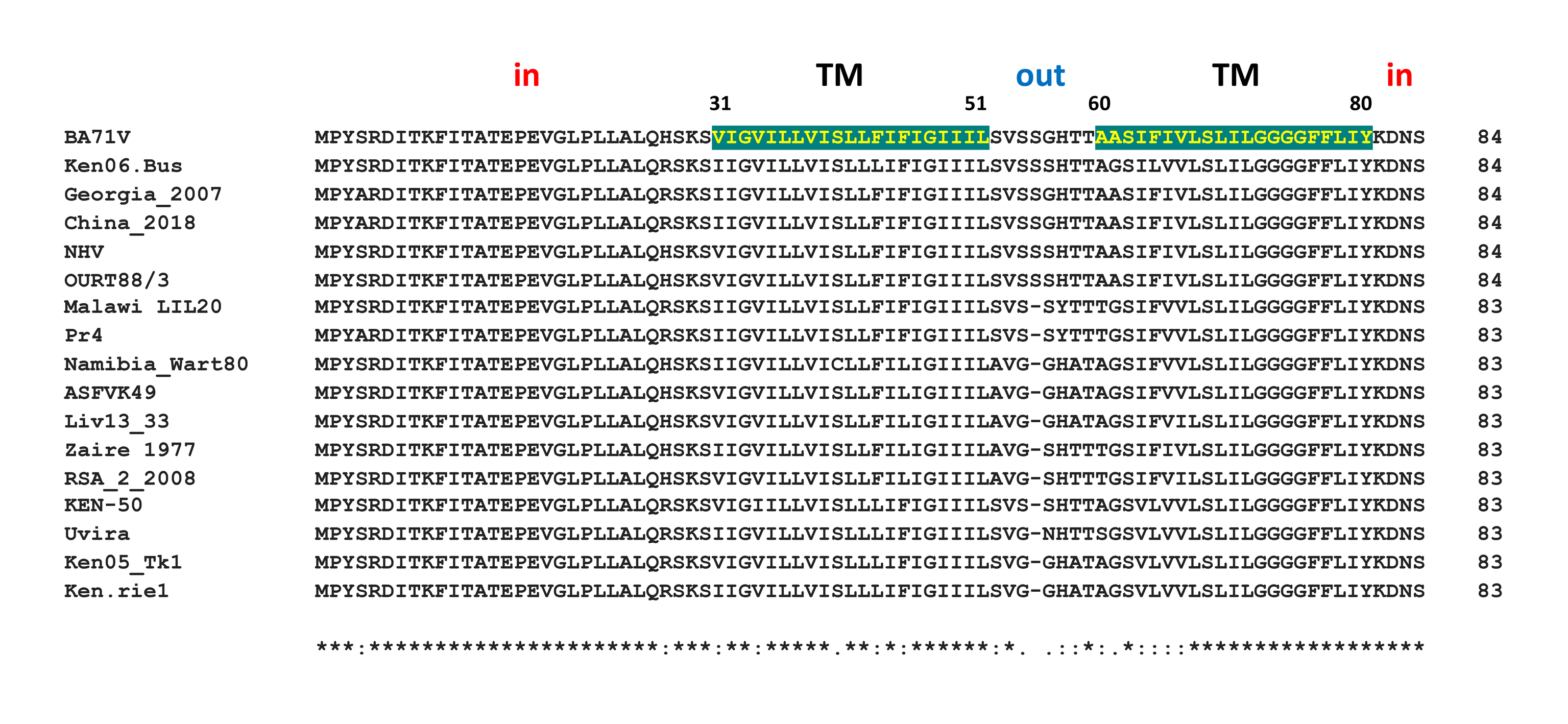

Supplement: S1 Fig — The aa sequences of pEP84R orthologues from different ASFV strains were retrieved from Genbank at the NCBI (https://www.ncbi.nlm.nih.gov/) and the corresponding accession numbers for each are: BA71V (NP_042748.1); Ken06.Bus (YP_009702953.1); Georgia_2007 (YP_009927178.1); China_2018(AYW34026.1); NHV (YP_009702621.1); OURT 88/3 (YP_009703662.1); Malawi LIL 20 (P0CAL5.1); Pr4 (P0CAL6.1); Namibia_Wart80 (P0CAL7.1); ASFVK49 (QZK26757.1); Liv13/33 (QID21215.1); Zaire 1977 (QII88576.1); RSA_2_2008 (QGM12834.2); KEN-50 (P0CAL4.1); Uvira (QRY19081.1); Ken05/Tk1 (YP_009702788.1); Ken.rie1 (CAD7112270.1). The sequences were aligned using Clustal Omega software at the EMBL-EBI (https://www.ebi.ac.uk/Tools/msa/clustalo/). Identical aa residues in all aligned sequences are designated with an asterisk at the bottom. Putative transmembrane regions in BA71V as annotated on the Uniprot website (https://www.uniprot.org/uniprot/Q07383) are indicated. The possible topology as predicted by TMHMM 2.0 at (https://services.healthtech.dtu.dk/service.php?TMHMM-2.0) is shown above the sequence, with “in” indicating the cytoplasmic side of the membrane. (TIF) [file ppat.1011136.s002.tif]

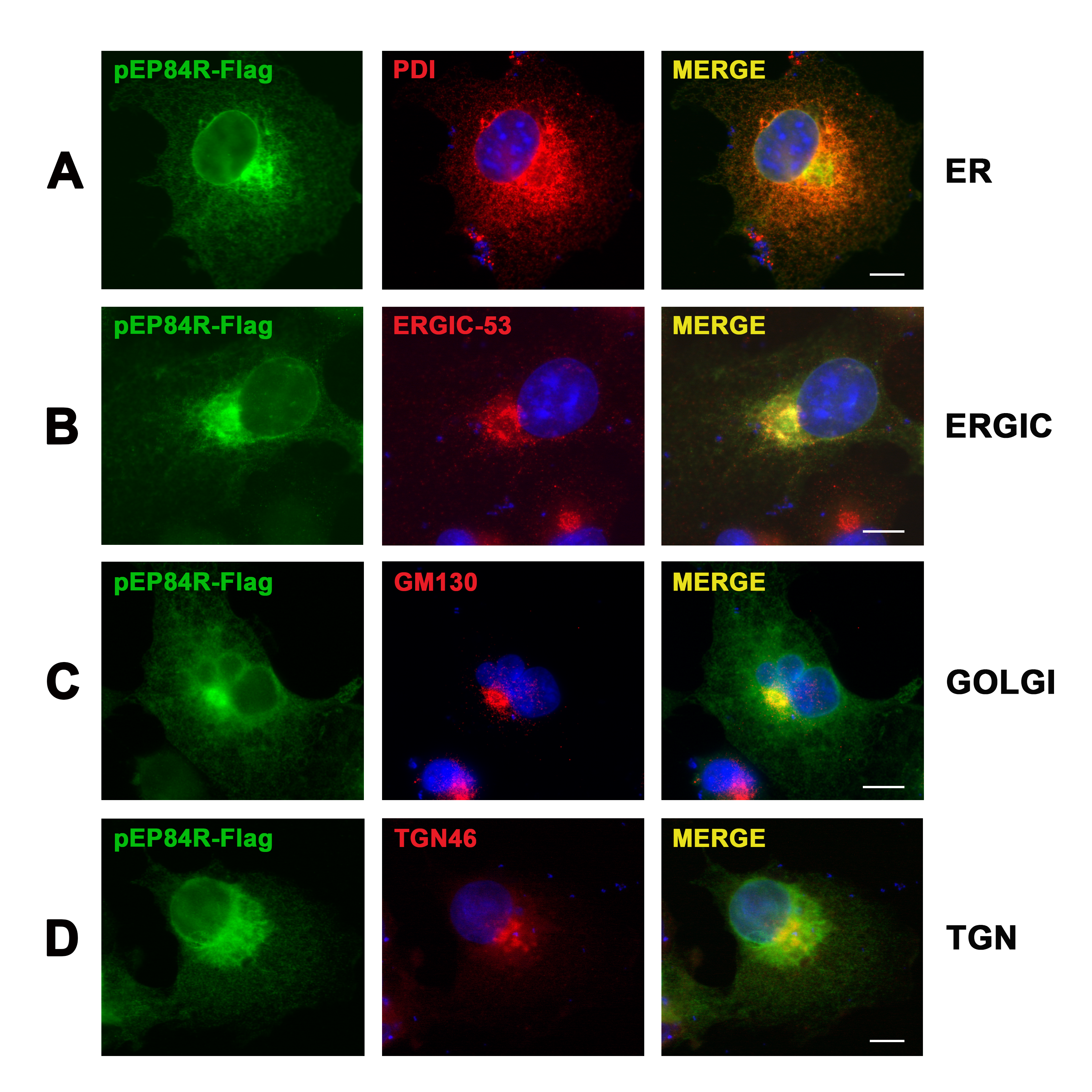

Supplement: S2 Fig — Cos cells were transfected with a plasmid containing EP84R gene fused to a C-terminal 3XFlag-tag epitope sequence (pEP84R-Flag). At 20 h, cells were fixed and immunolabelled with rabbit anti-pEP84R (A, B, C), or mAb anti-Flag (D), and antibodies against ER (PDI; A), ERGIC (ERGIC-53; B), cis-Golgi (GM130; C) and TGN (TGN-46; D) markers. Note the partial colocalization of pEP84R-Flag with PDI at the nuclear envelope and peripheral ER, and with ERGIC, Golgi complex and TGN markers at perinuclear areas. Bars, 10 μm. (TIF) [file ppat.1011136.s003.tif]

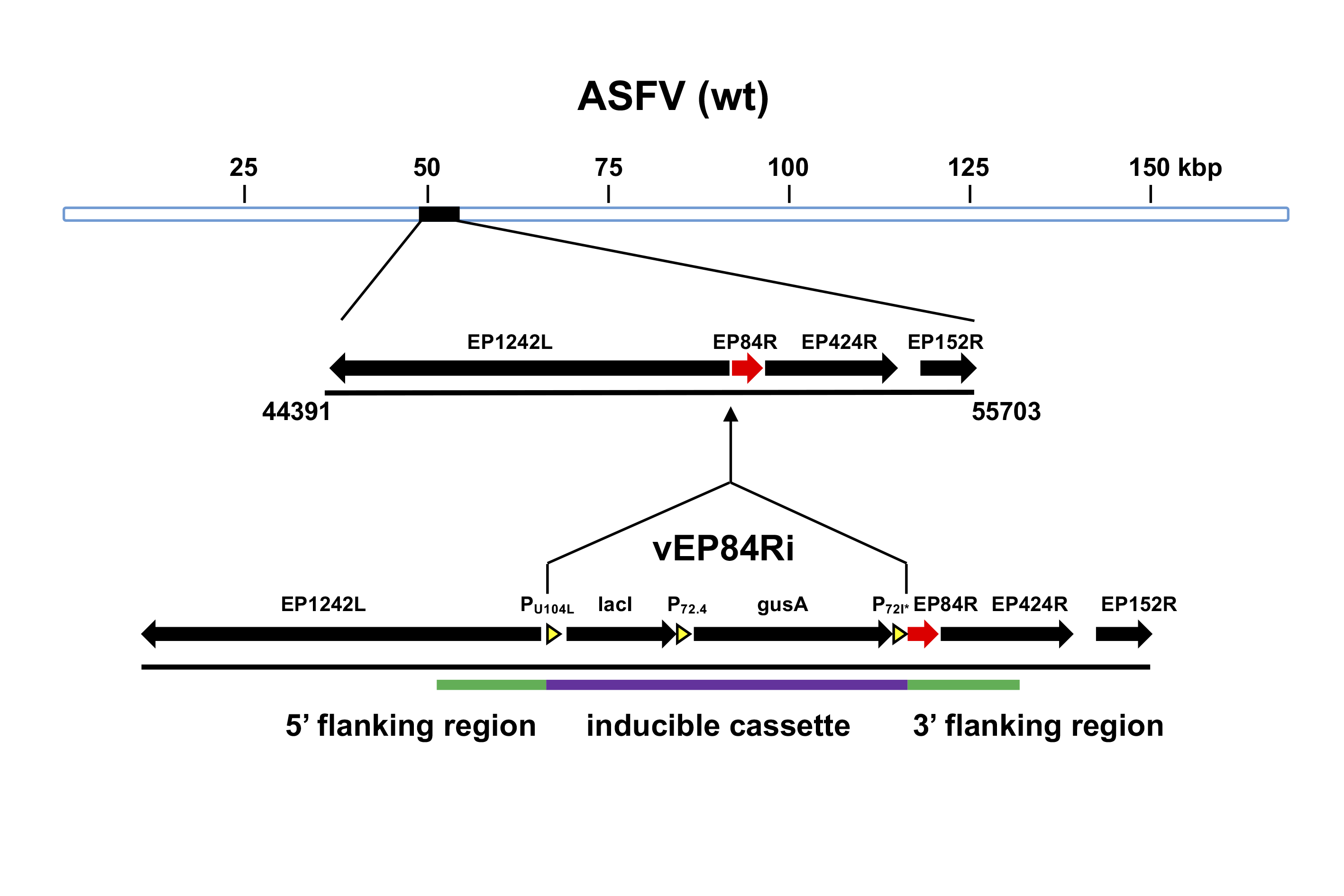

Supplement: S3 Fig — The recombinant virus was obtained by homologous recombination of parental ASFV genome (wt) with an inducible cassette containing a late, IPTG-dependent strong promoter (p72I*) for EP84R gene expression, a copy of E. coli lac repressor gene (lacI) and a reporter gene (gusA) used for selection and purification of the recombinant virus. ASFV genes in the proximity of the recombination area as well as the left and right flanking regions are indicated. (TIF) [file ppat.1011136.s004.tif]

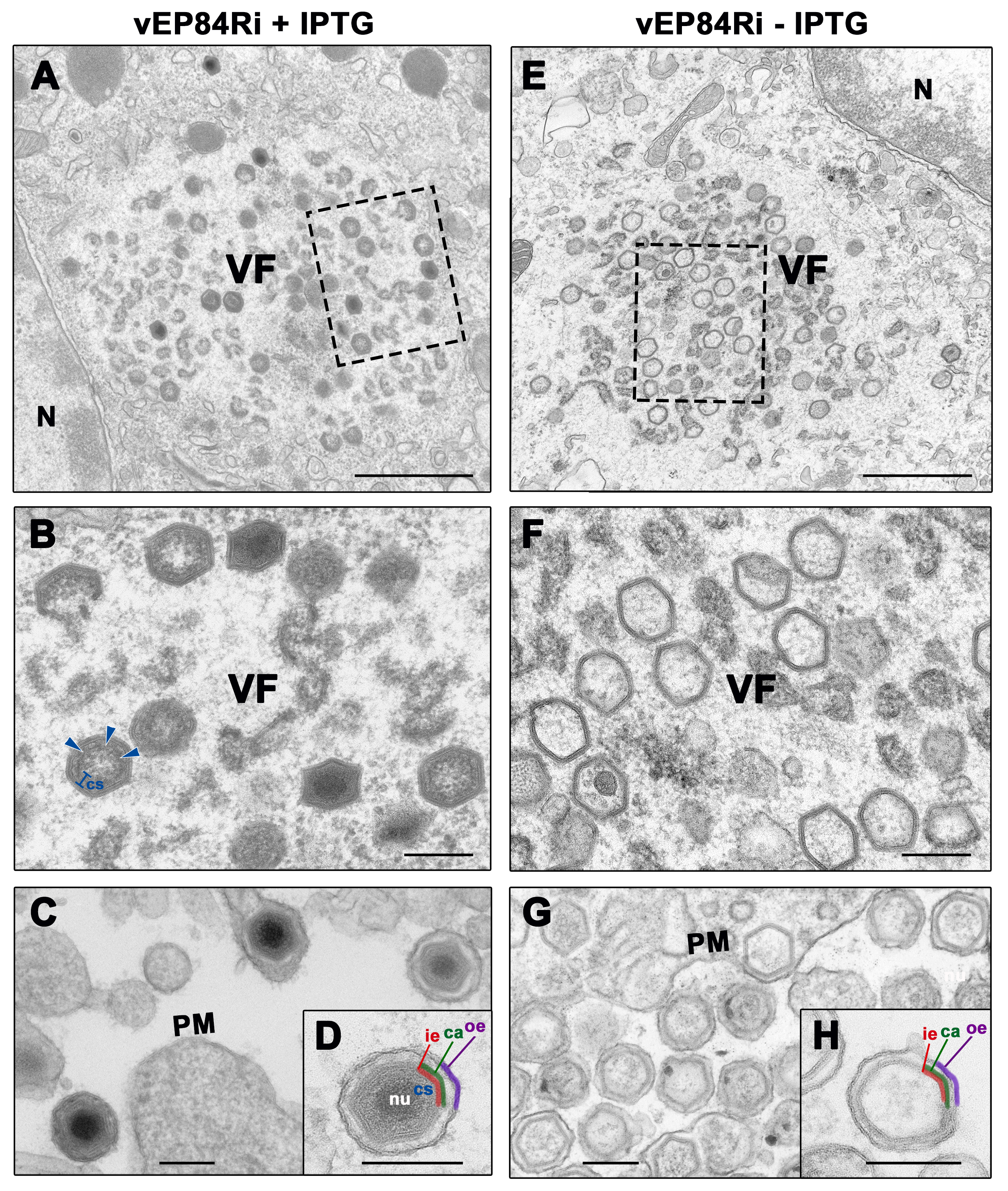

Supplement: S4 Fig — Porcine alveolar macrophages were infected with vEP84Ri for 18h in the presence (A-D) or in the absence (E-H) of IPTG. Panels B and F show higher magnification images of the viral factory areas delimited in A and E, respectively. Note that while under permissive conditions (+IPTG), the viral factories (VF) contain significant amounts of immature particles with a well-organized core shell (arrowheads in B) and mature virions, under non-permissive conditions (-IPTG), they contain essentially core-less particles (F). Note also that defective vEP84Ri- particles exit by budding from the PM (G-H), as occurs with ´full`vEP84Ri+ particles (C-D). The different virus layers (nucleoid (nu), core shell (cs), inner envelope (ie), capsid (ca) and outer envelope (oe)) of budding vEP84Ri+ (inset D) and vEP84Ri- (inset H) particles are indicated. Nucleus (N), plasma membrane (PM). Bars, 1 μm (A, E) and 200 nm (B-D and F-H). (TIF) [file ppat.1011136.s005.tif]

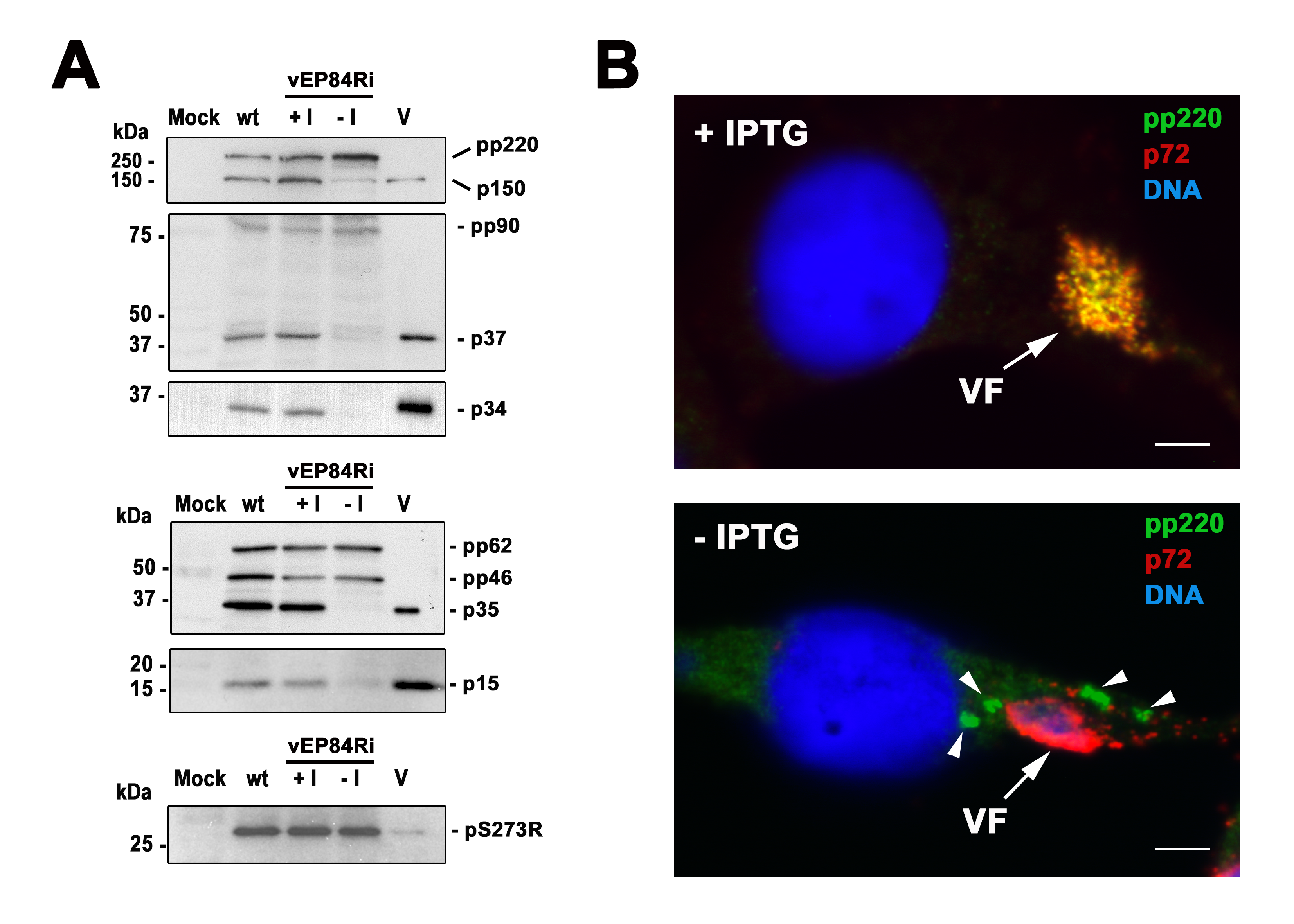

Supplement: S5 Fig — (A) Extracts of Vero cells mock-infected (Mock) or infected with parental BA71V (wt) or recombinant vEP84Ri viruses in the presence (+I) or absence (-I) of IPTG were analyzed by immunoblotting with antibodies against the mature products derived from polyprotein pp220 (upper) and pp62 (middle) and protease pS273R (bottom). As a control, purified ASFV particles (V) were also analyzed. Note that polyprotein processing is strongly impaired under restrictive conditions. The positions of polyprotein pp220 and pp62, the intermediate processing products pp90 and pp46, the mature products p150, p37, p34, p35 and p15, and the viral protease pS273R are indicated. Molecular masses are indicated on the left. (B) Immunofluorescence labeling of pp220 (green) and MCP p72 (red) in vEP84Ri-infected cells in the presence (+) or absence (-) of IPTG. Nuclear and viral DNA (blue) was stained with Hoechst 33258. The arrows indicate virus factories (VF) whereas the arrowheads indicate cytoplasmic pp220 accumulations outside the virus assembly sites under non-permissive conditions. Bars, 5 μm. (TIF) [file ppat.1011136.s006.tif]

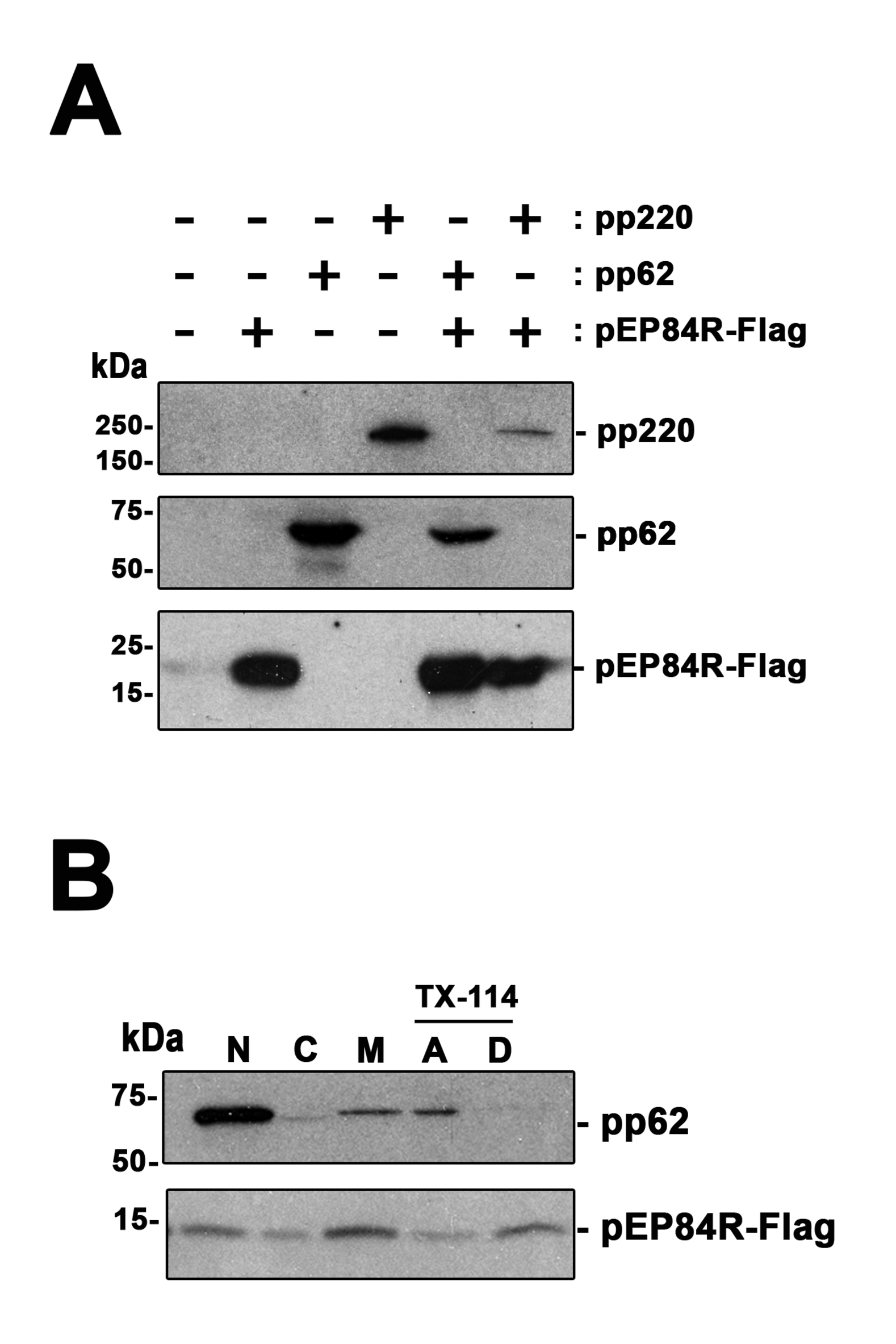

Supplement: S6 Fig — (A) Transfected Cos cells expressing proteins pEP84R-Flag, pp62 and pp220, individually or in combination, were analyzed by immunoblotting with antibodies to the target proteins. (B) Membrane-association of pEP84R-Flag and pp62. Transfected Cos cells expressing proteins pEP84R-Flag or pp62 were fractionated into cytosolic (C) and membrane/particulate (M) fractions. Also, the membrane fractions were subjected to TX-114 phase separation to obtain aqueous (A) and detergent-rich (D) phases. Equivalent fractions were analyzed by immunoblotting. Note that while pEP84R-Flag behaves as an integral membrane protein, pp62 behaves as a peripheral membrane protein. Molecular masses (in kDa) and pp220, pp62 and pEP84R-Flag bands are indicated. (TIF) [file ppat.1011136.s007.tif]

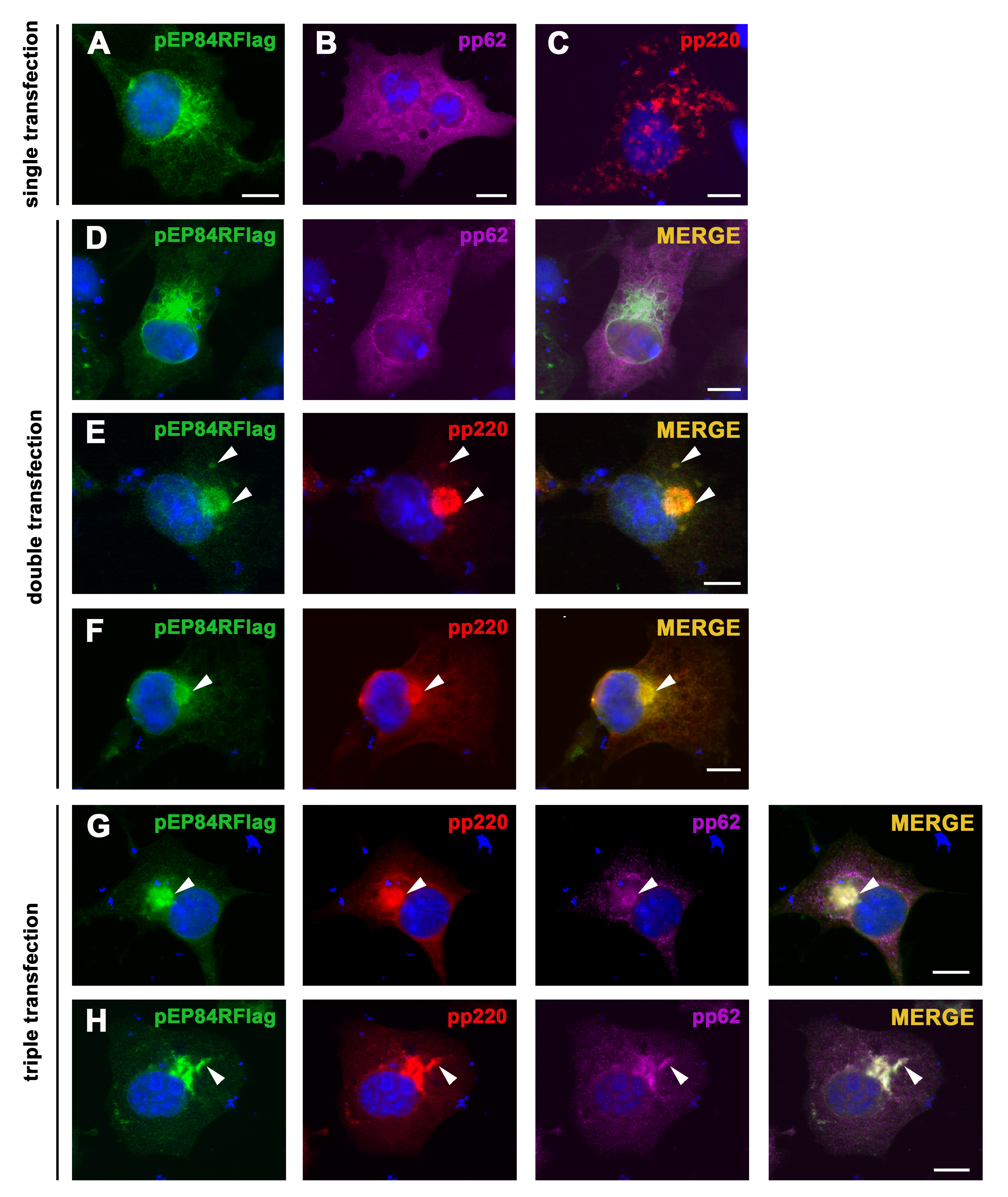

Supplement: S7 Fig — Transfected Cos cells expressing proteins pEP84R-Flag, pp62 and pp220 individually or in combination, were fixed and immunolabeled with rabbit antibodies to pEP84R (D, E, G and H), pp62 (C) and pp220 (B and F); mouse mAbs to pp62 (D, G and H), pp220 (E) and Flag tag (A and F) or rat antibody to pp220 (G and H) as indicated. Note that pp220 and pEP84R colocalize (E and F) to perinuclear areas and to a lesser extent to the cell surface (arrowheads). Note also the significant colocalization of pEP84R, pp220 and pp62 (G and H) at perinuclear areas (arrowheads). Bars, 10 μm. (TIF) [file ppat.1011136.s008.tif]

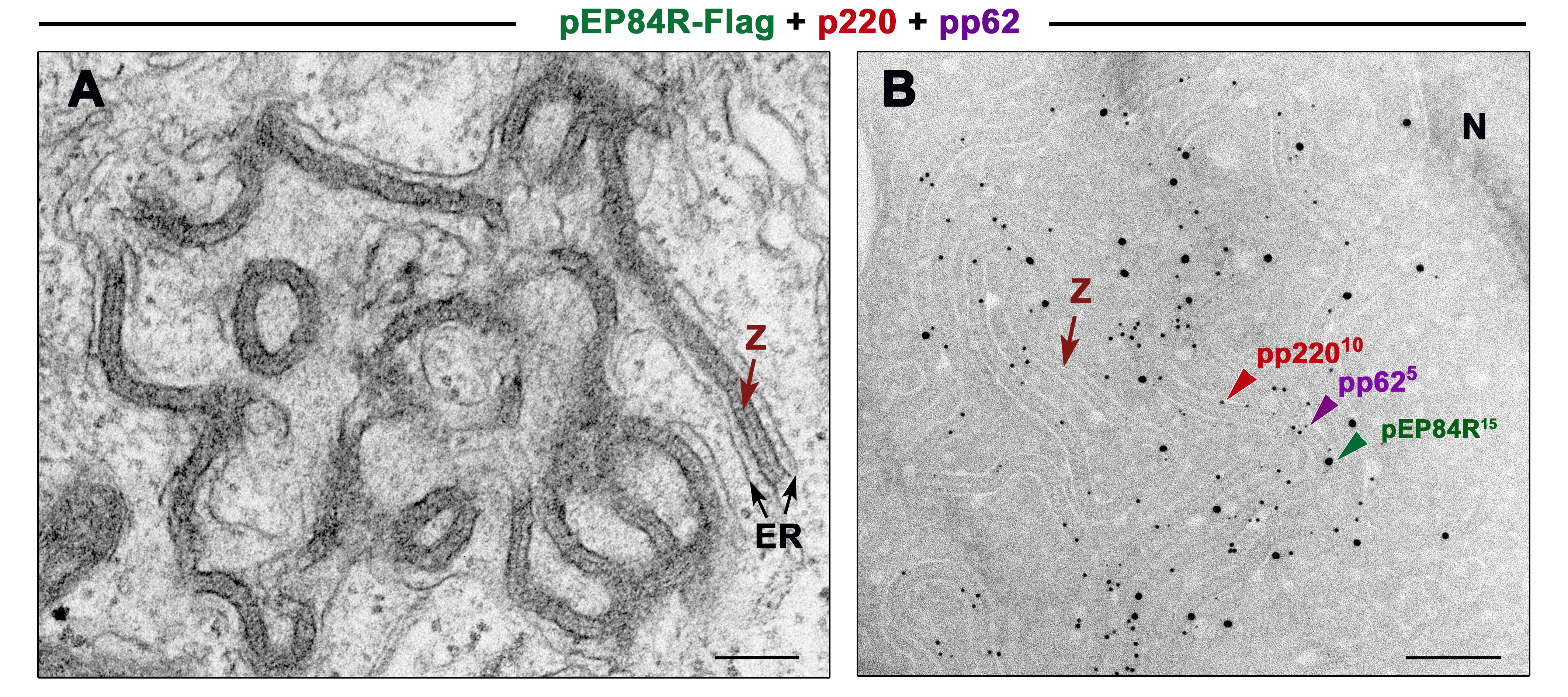

Supplement: S8 Fig — Transfected Cos cells co-expressing pEP84R-Flag, pp220 and pp62, were processed for conventional epoxy resin embedding (A) or cryosectioning and immunogold labeling (B) for pEP84R (15 nm, green arrowhead), pp220 (10 nm, red arrowhead) and pp62 (5 nm, purple arrowhead). Note the formation of zipper (z) structures associated to ER cisternae (A), which are immunolabeled for the three viral proteins (B). Bars, 200 nm. (TIF) [file ppat.1011136.s009.tif]

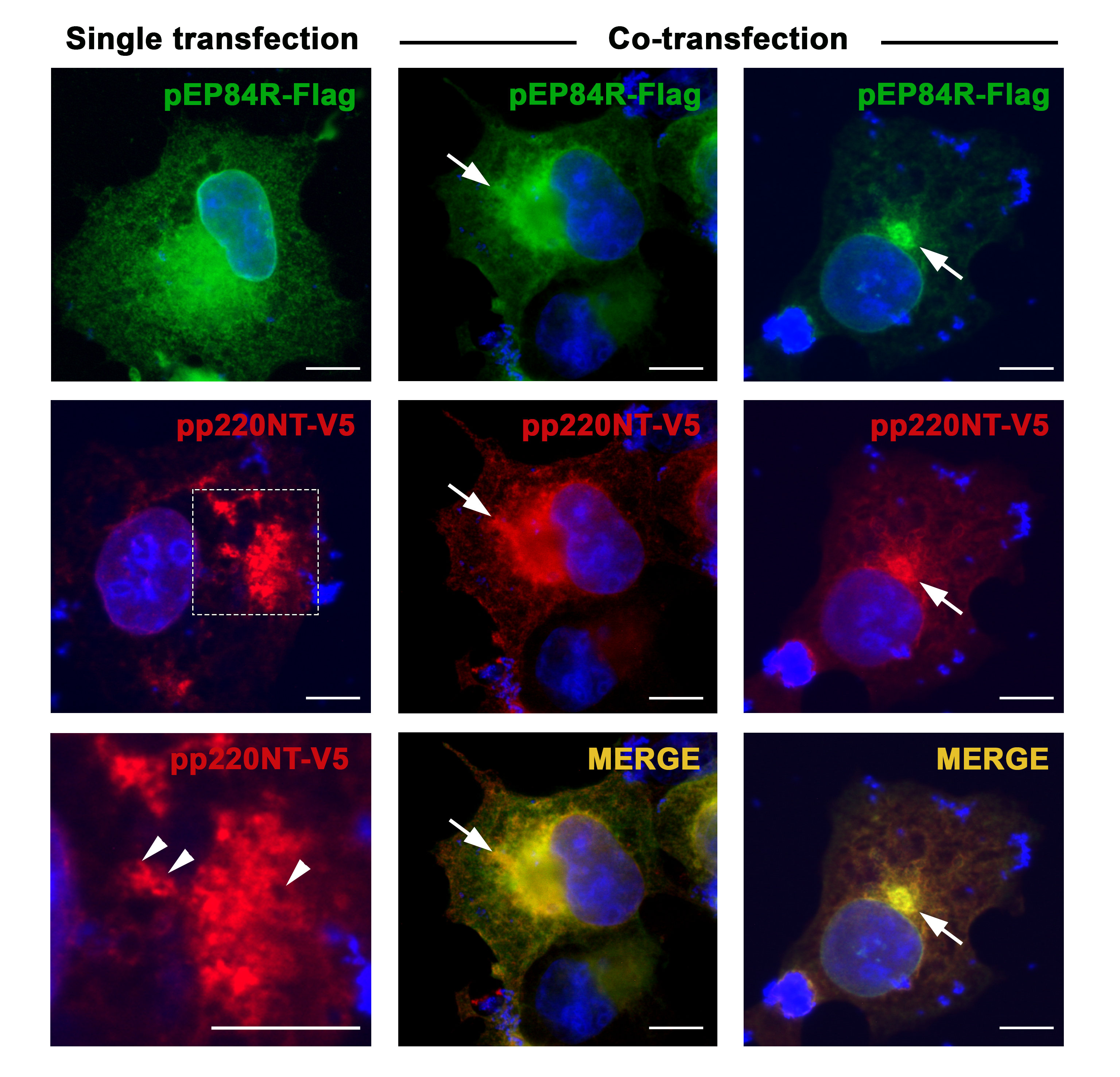

Supplement: S9 Fig — Cos cells transfected with pEP84R-Flag and pp220NT-V5 constructs individually or in combination, were immunolabeled with anti-pEP84R (green) and anti-V5 (red) antibodies. Note the vesicular-like pattern of pp220NT-V5 (middle left) indicated by arrowheads in a detail (bottom left) and colocalization (arrows in right panels) of pEP84R-Flag and pp220NT-V5 in co-transfected cells at perinuclear areas. Bars, 10 μm. (TIF) [file ppat.1011136.s010.tif]
